# Representation and Outcomes of Individuals With Schizophrenia Seen in Everyday Practice Who Are Ineligible for Randomized Clinical Trials

**DOI:** 10.1001/jamapsychiatry.2021.3990

**Published:** 2022-01-26

**Authors:** Heidi Taipale, Johannes Schneider-Thoma, Justo Pinzón-Espinosa, Joaquim Radua, Orestis Efthimiou, Christiaan H. Vinkers, Ellenor Mittendorfer-Rutz, Narcís Cardoner, Luis Pintor, Antti Tanskanen, Anneka Tomlinson, Paolo Fusar-Poli, Andrea Cipriani, Eduard Vieta, Stefan Leucht, Jari Tiihonen, Jurjen J. Luykx

**Affiliations:** 1 Department of Forensic Psychiatry, Niuvanniemi Hospital, University of Eastern Finland, Kuopio, Finland; 2 Divisions of Insurance Medicine, Department of Clinical Neuroscience, Karolinska Institutet, Stockholm, Sweden; 3 School of Pharmacy, University of Eastern Finland, Kuopio, Finland; 4 Department of Psychiatry and Psychotherapy, School of Medicine, Technical University of Munich, Munich, Germany; 5 Department of Mental Health, Parc Tauli University Hospital, Sabadell, Barcelona, Spain; 6 Department of Medicine, University of Barcelona School of Medicine, Barcelona, Spain; 7 Department of Clinical Psychiatry, University of Panama School of Medicine, Panama City, Panama; 8 Institut d’Investigacions Biomèdiques August Pi i Sunyer (IDIBAPS), CIBERSAM, Barcelona, Spain; 9 Early Psychosis: Interventions and Clinical-detection (EPIC) Lab, Department of Psychosis Studies, Institute of Psychiatry, Psychology, and Neuroscience, King’s College London, London, United Kingdom; 10 Institute of Social and Preventive Medicine, University of Bern, Bern, Switzerland; 11 Department of Psychiatry, University of Oxford, Oxford, United Kingdom; 12 Department of Psychiatry, Amsterdam Neuroscience, Amsterdam UMC, Vrije Universiteit Amsterdam, Amsterdam, the Netherlands; 13 Institut d’Investigació I Innovació Parc Tauli (I3PT), CIBERSAM, Sabadell, Barcelona, Spain; 14 Department of Psychiatry and Forensic Medicine, Universitat Autònoma de Barcelona, Barcelona, Spain; 15 Department of Psychiatry and Psychology, Hospital Clinic, University of Barcelona, Barcelona, Spain; 16 Oxford Health NHS Foundation Trust, Warneford Hospital, Oxford, United Kingdom; 17 Department of Brain and Behavioral Sciences, University of Pavia, Pavia, Italy; 18 OASIS Service, South London and Maudsley NHS Foundation Trust, London, United Kingdom; 19 Center for Psychiatry Research, Stockholm City Council, Stockholm, Sweden; 20 Department of Psychiatry, UMC Utrecht Brain Center, University Medical Center Utrecht, Utrecht University, Utrecht, the Netherlands; 21 Department of Psychiatry and Neuropsychology, School for Mental Health and Neuroscience, Maastricht University Medical Centre, Maastricht, the Netherlands; 22 Outpatient second opinion clinic, GGNet Mental Health, Warnsveld, the Netherlands

## Abstract

**Importance:**

Most evidence about efficacy and safety of antipsychotics in schizophrenia spectrum disorders relies on randomized clinical trials (RCTs). However, owing to their strict eligibility criteria, RCTs represent only a part of the real-world population (ie, unselected patients seen in everyday clinical practice), which may result in an efficacy-effectiveness gap.

**Objective:**

To quantify the proportion of real-world individuals with schizophrenia spectrum disorders who would be ineligible for participation in RCTs, and to explore whether clinical outcomes differ between eligible and ineligible individuals.

**Design, Setting, and Participants:**

This study applied eligibility criteria typically used in RCTs for relapse prevention in schizophrenia spectrum disorders to real-world populations. Individuals with diagnoses of schizophrenia spectrum disorders recorded in national patient registries in Finland and Sweden were identified. Individuals who had used antipsychotics continuously for 12 weeks in outpatient care were selected. Individuals were followed up for up to 1 year while they were receiving maintenance treatment with any second-generation antipsychotic (excluding clozapine). Follow-up was censored at treatment discontinuation, initiation of add-on antipsychotics, death, and end of database linkage.

**Main Outcomes and Measures:**

Proportions of RCT-ineligible individuals with schizophrenia spectrum disorders owing to any and specific RCT exclusion criteria. The risk of hospitalization due to psychosis within 1-year follow-up in ineligible vs eligible persons were compared using hazard ratios (HR) and corresponding 95% CIs.

**Results:**

The mean (SD) age in the Finnish cohort (n = 17 801) was 47.5 (13.8) years and 8972 (50.4%) were women; the mean (SD) age in the Swedish cohort (n = 7458) was 44.8 (12.5) years and 3344 (44.8%) were women. A total of 20 060 individuals (79%) with schizophrenia spectrum disorders would be ineligible for RCTs (Finnish cohort: 14 221 of 17 801 [79.9%]; Swedish cohort: 5839 of 7458 [78.3%]). Most frequent reasons for ineligibility were serious somatic comorbidities and concomitant antidepressant/mood stabilizer use. Risks of hospitalization due to psychosis was higher among ineligible than eligible individuals (Finnish cohort: 18.4% vs 17.2%; HR, 1.14 [95% CI, 1.04-1.24]; Swedish cohort: 20.1% vs 14.8%; HR, 1.47 [95% CI, 1.28-1.92]). The largest risks of hospitalization due to psychosis were observed in individuals ineligible owing to treatment resistance, tardive dyskinesia, and history of suicide attempts. Finally, with more ineligibility criteria met, larger risks of hospitalization due to psychosis were observed in both countries.

**Conclusions and Relevance:**

RCTs may represent only about a fifth of real-world individuals with schizophrenia spectrum disorders. Underrepresented (ineligible) patients with schizophrenia spectrum disorders have moderately higher risks of admission due to psychosis while receiving maintenance treatment than RCT-eligible patients. These findings set the stage for future studies targeting real-world populations currently not represented by RCTs.

## Key Points

**Question:**

What percentage of patients with schizophrenia in the real world are represented in randomized clinical trials (RCTs) and do their outcomes differ from those not represented in RCTs?

**Findings:**

In this study of 25 259 real-world individuals with diagnoses of schizophrenia spectrum disorders recorded in national patient registries in Finland and Sweden, about a fifth were represented in RCTs and their outcomes were better than of those individuals with schizophrenia not meeting RCT inclusion criteria.

**Meaning:**

Future research should consider the heterogeneity of individuals with schizophrenia and the patient groups typically ineligible for participation; RCTs may become more inclusive by representing a broader spectrum of individuals with schizophrenia and by targeting specific currently underrepresented groups.

## Teaser

This cohort study quantifies the proportion of real-world individuals with schizophrenia spectrum disorders who would be ineligible for participation in randomized clinical trials and explores whether clinical outcomes differ between eligible and ineligible individuals.

## Introduction

Most evidence about efficacy and safety of medical treatments is based on randomized clinical trials (RCTs), which are highly standardized, systematic studies. RCT outcomes (efficacy) may differ from the utility of interventions in routine clinical practice (effectiveness), in what has been termed the efficacy-effectiveness gap. Efficacy-effectiveness gaps have been identified in several health care areas, including pneumology,^1,2^ oncology,^3^ infectology,^4^ and internal medicine^5^; nonpharmacological interventions in psychology^6^; and antidepressants.^7^

A possible efficacy-effectiveness gap in effectiveness and safety of antipsychotics in individuals with schizophrenia, which, to our knowledge, has not been investigated so far, may stem from the strict exclusion criteria applied in typical RCTs aiming at marketing approval. Therefore, a broad and diverse set of individuals is excluded from these trials, such as those experiencing suicidal ideations, substance use disorders, or somatic and psychiatric comorbidities. Such excluded people may have different courses of illness and possibly also different treatment outcomes.

Here, we aimed to quantify the real-world population (ie, unselected patients seen in everyday clinical practice) not directly represented in RCTs, ie, those who are ineligible owing to any RCT exclusion criteria, as well as the real-world populations ineligible owing to specific exclusion criteria. Moreover, we assessed whether there are differences in key outcomes between individuals who are potentially eligible and those who are not (overall and for specific RCT exclusion criteria). To answer these research questions, we analyzed data from real-world populations in 2 nationwide registries.

## Methods

In this analysis, we simulated the application of typical inclusion and exclusion criteria of RCTs conducted in individuals with schizophrenia (eAppendix 1 in the Supplement) to the national patient registries of Finland and Sweden. The protocol for our analysis was registered on the Open-Science Framework prior to analysis on September 15, 2020,^8^ and we complied with the Reporting of Studies Conducted Using Observational Routinely-Collected Data (RECORD) reporting guideline (eAppendix 2 in the Supplement).^9^ The Regional Ethics Board of Stockholm approved this research project (decision 2007/762–31). Permissions were also granted by pertinent institutional authorities at the Finnish National Institute for Health and Welfare (permission THL/847/5.05.00/2015), the Social Insurance Institution of Finland (65/522/2015), and Statistics Finland (TK53-1042-15). The study was registry based, and no contact was made with the participants of the study; therefore, according to legislation in both countries, obtaining informed consent from participants was not required.

### Real-World Databases Used and Follow-up

We had access to the data extracted from the national patient registries in Finland (January 2005-December 2017) and Sweden (January 2006-December 2016) (eAppendix 1A in the Supplement includes details about the cohorts), which here represent real-world individuals with schizophrenia and schizoaffective disorder (referred to from here on as *schizophrenia*). Pseudonymized data were originally extracted by the register maintainers via personal identity codes, which enable data linkage between registries of both countries. Personal identity codes were replaced with research identity codes before data were shared with the researchers. We chose 2 registries to assess similarity in findings across countries and reduce the likelihood of chance findings.

In both registries, we first focused on individuals hospitalized at least once owing to schizophrenia and who used second-generation antipsychotics at the start of follow-up because those are the typical interventions in modern RCTs^10^ and also the most used antipsychotics in Finland and Sweden nowadays.^11^ We did not consider individuals using clozapine or first-generation antipsychotics because the former is not a first-line treatment but reserved for treatment resistance (here defined as clozapine or electroconvulsive therapy treatment, reported ever before follow-up), and the latter are only rarely used in real-world clinical practice in Finland and Sweden.^11^ Continuous medication use was derived using the PRE2DUP method from dispensed prescriptions.^12^

The duration of follow-up was 12 months as this is a typical duration of relapse-prevention RCTs,^10^ with time zero defined as when the inclusion criteria were fulfilled, ie, after 12 weeks of continuous antipsychotic use in monotherapy as an outpatient. We chose this 12-week criterion to ensure clinical stability of schizophrenia in maintenance treatment with antipsychotics, which is a starting point for relapse prevention trials. We censored follow-up at discontinuation of antipsychotics, hospitalization (other types than analyzed as outcome event), death, after the defined follow-up time, and end of data linkage. For details about patient involvement and data sharing options, see the eMethods in the Supplement.

### Outcomes and Statistical Analyses

By applying to these databases the standard RCT inclusion and exclusion criteria mentioned in the eMethods (eAppendix 1A) in the Supplement, we defined populations consisting of:

1. Individuals potentially eligible for standard RCTs about relapse prevention with antipsychotics (ie, meeting all inclusion criteria but having none of the exclusion criteria);
2. Individuals ineligible for such an RCT for any reason (ie, meeting all inclusion criteria but having ≥1 exclusion criteria);
3. Individuals ineligible for such an RCT owing to each specific exclusion criterion (ie, forming subpopulations of ineligible individuals owing to age, substance use, risk of suicide, treatment resistance, serious somatic disease, mood stabilizer or antidepressant use, intellectual disability, tardive dyskinesia, or pregnancy/breastfeeding).

We summarized relevant baseline characteristics and report the distribution of the prescribed antipsychotics (for the most commonly used drugs)^11^ of the eligible and ineligible populations. Based on previous knowledge,^11^ we categorized most commonly prescribed antipsychotics in these cohorts as olanzapine, quetiapine, risperidone, and aripiprazole, while the rest were grouped as either any long-acting injectable (LAI) antipsychotic or other oral antipsychotics.

The primary outcome was hospitalization due to psychosis (*International Statistical Classification of Diseases and Related Health Problems, Tenth Revision *codes F20-F29). Secondary outcomes were hospitalization due to any psychiatric reason (*International Statistical Classification of Diseases and Related Health Problems, Tenth Revision *codes F00-F99), all-cause hospitalization, the need for add-on antipsychotics, and all-cause discontinuation of antipsychotic use. To verify the robustness of our results for different time points, in addition to the main analyses at 12 months, we conducted analyses for the primary outcome at 6 months and 9 months of follow-up. Additionally, as sensitivity analyses, we applied the same primary outcome analyses to separate cohorts of (1) clozapine users and (2) individuals only treated in outpatient care (eAppendix 1A in the Supplement).

To compare potential differences in the risk of these outcomes between eligible and ineligible individuals, we calculated hazard ratios (HRs) and their 95% CIs using a Cox regression model with eligible individuals as reference. Proportional hazards assumption was tested and complied with by plotting Kaplan-Meier curves and via Schoenfeld residuals. To shed light on the associations of specific exclusion criteria with the primary outcome, we additionally compared individuals with a given exclusion criterion with eligible individuals. We also compared the primary outcome between individuals who met 1, 2, or 3 or more exclusion criteria with those of eligible individuals. We conducted all analyses using SAS statistical software version 9.4 (SAS Institute) between November 2020 and May 2021. We calculated 95% CIs to provide estimates of the accuracy of our population parameters. *P* values are only reported in Tables as additional measures of the magnitude and precision of the differences, but we do not characterize results as statistically significant or according to some arbitrary *P* value threshold.

## Results

### Proportions, Descriptive Statistics, and Antipsychotic Use in Eligible and Ineligible Populations

The mean (SD) age in the Finnish cohort (n = 17 801) was 47.5 (13.8) years, and 8972 (50.4%) were women; the mean (SD) age in the Swedish cohort (n = 7458) was 44.8 (12.5) years, and 3344 (44.8%) were women. In the Finnish cohort, 3580 individuals (20.1%) were eligible for RCT participation; 14 221 (79.9%) met at least 1 exclusion criterion and were thus ineligible. Similarly, in the Swedish cohort, 1619 individuals (21.7%) were eligible for RCTs, and 5839 (78.3%) were ineligible.

There were no major differences in the distribution of age and sex between eligible and ineligible individuals (Table 1). Individuals who were ineligible for RCTs were more likely to use oral quetiapine (Finland: 3735 [26.3%] vs 612 [17.1%]; Sweden: 809 [13.9%] vs 110 [6.8%]; eFigure 1 in the Supplement). LAI antipsychotics were prescribed less frequently to ineligible than to eligible individuals (Finland: 1767 [12.4%] vs 753 [21.0%]; Sweden: 1075 [18.4%] vs 390 [24.1%]; eFigure 1 in the Supplement). In the Swedish data set where information on disability pension was available, ineligible individuals were somewhat more likely (4985 [85.4%]) to receive disability pension than eligible ones (1320 [81.5%]), indicating more severe decline in occupational function.

**Table 1.** Characteristics of Individuals Included (Individuals Without Any Exclusion Criteria) vs Excluded (After Application Of All Exclusion Criteria) for Randomized Clinical Trials

Characteristic	No. (%)			
	Finnish cohort (n = 17 801)		Swedish cohort (n = 7458)	
	Eligible (n = 3580)	Ineligible (n = 14 221)	Eligible (n = 1619)	Ineligible (n = 5839)
Male	1837 (51.3)	6992 (49.2)	962 (59.4)	3152 (54.0)
Female	1743 (48.7)	7229 (50.8)	657 (40.6)	2687 (46.0)
Age, y				
<18	0	26 (0.2)	0	9 (0.2)
18-30	676 (18.9)	2470 (17.4)	205 (12.7)	770 (13.2)
31-45	1016 (28.4)	3624 (25.5)	627 (38.7)	1892 (32.4)
46-65	1888 (52.7)	6332 (44.5)	787 (48.6)	2774 (47.5)
>65	0	1769 (12.4)	0	394 (6.8)
Age, mean (SD), y	45.6 (12.3)	47.9 (14.1)	44.9 (11.2)	46.5 (12.8)
Schizoaffective disorder	385 (10.8)	3459 (24.3)	232 (14.3)	2045 (35.0)
Disability pension	NA	NA	1320 (81.5)	4985 (85.4)

Abbreviation: NA, not applicable.

### Ineligibility Reasons and Subpopulations

In the Finnish and Swedish cohorts, 5875 (33.0%) and 2514 (33.7%), respectively, fulfilled only 1 exclusion criterion, while 3271 (18.4%) and 1338 (17.9%), respectively, met 3 or more criteria (Figure, A). The most frequent reasons for ineligibility were serious somatic comorbidities (broad definition: 7202 [51%] and 2866 [49%]; narrow: 5287 [36%] and 1747 [30%] in Finland and Sweden, respectively) and concomitant use of mood stabilizers or antidepressants (7983 [56%] and 3281 [56%]), followed by a history of substance use (3808 [27%] and 1828 [31%]) and suicide risk (1690 [12%] and 1032 [18%]) (Figure, B).

**Figure.**
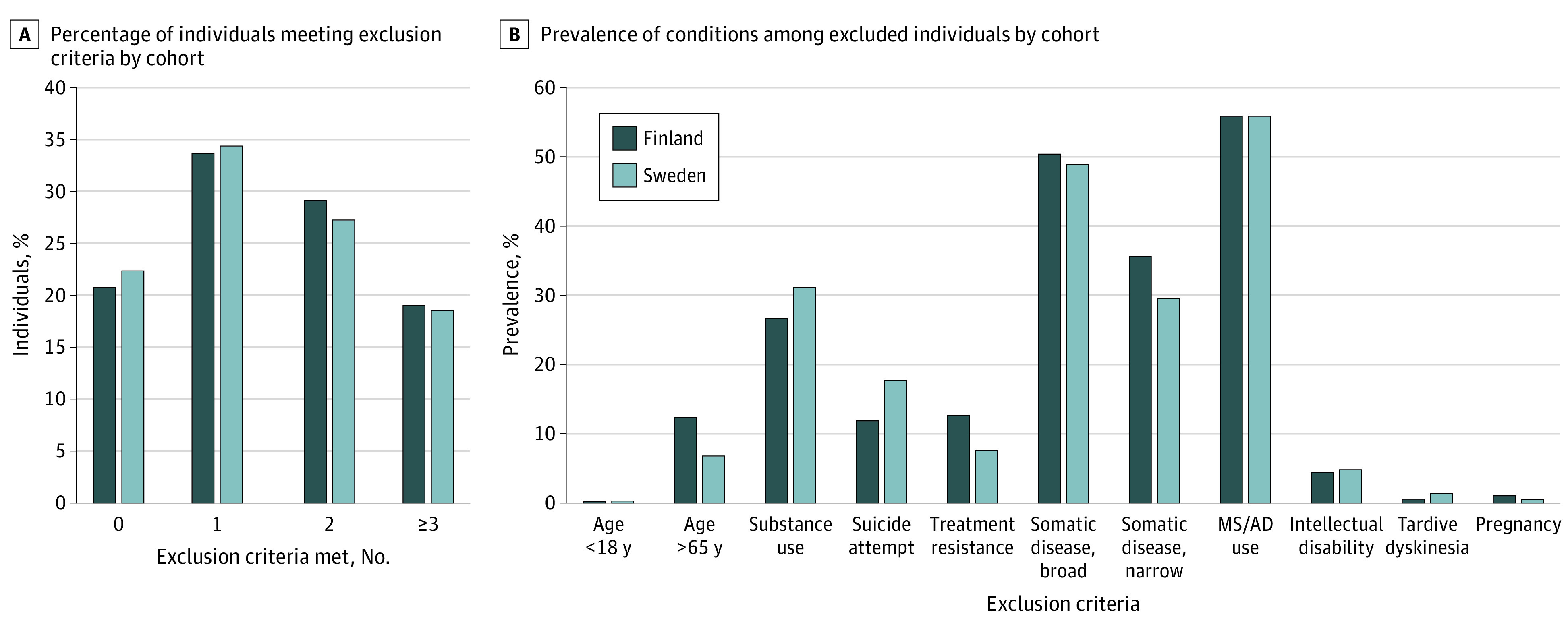
Distribution of the Number of Exclusion Criteria Met and Prevalence of Specific Conditions Among Persons Ineligible for Randomized Clinical Trials A, Percentage of individuals in the cohorts who fulfilled none, 1, 2, or ≥3 exclusion criteria in the Finnish and Swedish cohorts. B, Prevalence (%) of specific conditions among individuals ineligible for randomized clinical trials in the Finnish and Swedish cohorts. AD indicates antidepressant; MS, mood stabilizer.

Subpopulations ineligible owing to specific exclusion criteria had variation in age and sex distributions (eTable 1A and B in the Supplement). The proportion of men was highest in those excluded owing to substance use (2510 men [65.9%] in the Finnish cohort and 1230 [67.3%] in the Swedish cohort), whereas those excluded owing to age (almost entirely owing to age >65 years) were mainly women (687 men [38.3%] in the subgroup of the Finnish cohort excluded owing to age and 143 [36.3%] in the Swedish cohort). There was significant overlap between specific exclusion criteria with each other. Besides obvious overlap between broad and narrow definitions of serious somatic comorbidities, those with a history of suicide attempt often also had substance use (Finnish cohort: 882 [52%]; Swedish cohort: 533 [52%]) and mood stabilizers/antidepressants use (1009 [58%] in the Finnish and 603 [60%] in the Swedish cohorts) and those with tardive dyskinesia had serious somatic conditions (24 [60%] and 47 [63%] in the Finnish and Swedish cohorts, respectively) and mood stabilizers/antidepressants use (24 [60%] and 36 [48%] in the Finnish and Swedish cohorts, respectively).

Olanzapine was the most frequently prescribed antipsychotic across specific subpopulations (eFigures 2 and 3 in the Supplement). Quetiapine replaced olanzapine as the most frequently prescribed antipsychotic among those with a history of suicide attempts and among those with tardive dyskinesia in the Finnish cohort. Risperidone was equally commonly prescribed among pregnant or breastfeeding individuals as olanzapine in the Swedish cohort. In Finland, LAI antipsychotics were used in about 10% of all individuals in most subpopulations (except substance use), while in Sweden the use of LAI antipsychotics was more frequent: almost 20% in all groups (except mood stabilizers/antidepressants users).

### Primary and Secondary Outcomes in Eligible and Ineligible Populations

During the 12 months of follow-up, individuals who would be ineligible for RCTs were more likely to be hospitalized owing to psychosis, compared with eligible individuals (Finnish cohort: 2609 [18.4%] vs 615 [17.2%]; HR, 1.14 [95% CI, 1.04-1.24]; Swedish cohort: 1174 [20.1%] vs 240 [14.8%]; HR, 1.47 [95% CI, 1.28-1.92]). Similar risk estimates were observed for 6- and 9-month follow-up times (Table 2).

**Table 2.** Risk of Hospitalization Due to Psychosis in Individuals Ineligible (After Application of ≥1 Exclusion Criteria) vs Eligible (Persons Without Any Exclusion Criterion)

Eligibility	Finnish cohort					Swedish cohort				
	No. of individuals	No. (%) with event	Time to event/censoring, mean (SD), d	HR (95% CI)^a^	*P* value	No. of individuals	No. (%) with event	Time to event/censoring, mean (SD), d	HR (95% CI)^a^	*P* value
**Main outcome analyses: hospitalization due to psychosis, 12 mo**										
Eligible	3580	615 (17.2)	278 (130)	1.14 (1.04-1.24)	.005	1619	240 (14.8)	273 (127)	1.47 (1.28-1.92)	<.001
Ineligible	14 221	2609 (18.4)	257 (137)			5839	1174 (20.1)	248 (137)		
**Alternative time period 1: hospitalization due to psychosis, 6 mo**										
Eligible	3580	443 (12.4)	154 (56)	1.15 (1.03-1.27)	.01	1619	166 (10.3)	156 (53)	1.49 (1.26-1.75)	<.001
Ineligible	14 221	1939 (13.6)	147 (60)			5839	835 (14.3)	145 (60)		
**Alternative time period 2: hospitalization due to psychosis, 9 mo**										
Eligible	3580	537 (15.0)	218 (93)	1.15 (1.05-1.26)	.004	1619	212 (13.1)	218 (89)	1.45 (1.25-1.69)	<.001
Ineligible	14 221	2327 (16.4)	205 (98)			5839	1033 (17.7)	200 (99)		

Abbreviation: HR, hazard ratio.

^a^ An HR >1 means higher risk in the ineligible group. Primary analyses were with 12-month follow-up and sensitivity analyses with 6 and 9 months.

Compared with eligible individuals, individuals who were ineligible for RCTs had increased risks of any psychiatric hospitalization (HR, 1.34 [95% CI, 1.23-1.45]) in the Finnish cohort; HR, 1.74 [95% CI, 1.52-1.99] in the Swedish cohort) and for all-cause hospitalization (HR, 1.55 [95% CI, 1.43-1.68] in the Finnish cohort; HR, 1.77 [95% CI, 1.55-2.03] in the Swedish cohort; Table 3). In the Swedish cohort, ineligible persons had a higher risk for needing an additional antipsychotic than eligible persons (HR, 1.31 [95% CI, 1.15-1.48]), which was not observed in the Finnish cohort (HR, 1.06 [95% CI, 0.96-1.17]). The risk of all-cause antipsychotic discontinuation did not differ between ineligible and eligible individuals (Table 3).

**Table 3.** Risk of Secondary Outcomes in Individuals Ineligible (After Application of ≥1 Exclusion Criteria) vs Eligible (Individuals Without Any Exclusion Criteria) at 12-Month Follow-up

Eligibility	Finnish cohort				Swedish cohort			
	No. (%) with event	Time to event/censoring, mean (SD)	HR (95% CI)^a^	*P* value	No. (%) with event	Time to event/censoring, mean (SD)	HR (95% CI)^a^	*P* value
**Hospitalization due to any psychiatric reason**								
Eligible	643 (18.0)	278 (131)	1.34 (1.23-1.45)	<.001	251 (15.5)	273 (127)	1.74 (1.52-1.99)	<.001
Ineligible	3202 (22.5)	256 (138)			1440 (24.7)	246 (138)		
**All-cause hospitalization**								
Eligible	694 (19.4)	280 (130)	1.55 (1.43-1.68)	<.001	250 (15.4)	273 (127)	1.77 (1.55-2.03)	<.001
Ineligible	4011 (28.2)	259 (136)			1478 (25.31)	246 (138)		
**Need for additional antipsychotic**								
Eligible	454 (12.7)	254 (138)	1.06 (0.96-1.17)	.28	286 (17.7)	238 (137)	1.31 (1.15-1.48)	<.001
Ineligible	1783 (12.5)	233 (141)			1221 (20.9)	211 (141)		
**All-cause discontinuation of antipsychotic use**								
Eligible	570 (15.9)	280 (130)	1.03 (0.94-1.13)	.59	408 (25.2)	275 (126)	1.01 (0.90-1.12)	.91
Ineligible	2191 (15.4)	259 (137)			1370 (23.5)	251 (136)		

Abbreviation: HR, hazard ratio.

^a^ An HR >1 means higher risk in the ineligible group.

### Primary Outcome in Subpopulations Ineligible for Specific Reasons

The largest risks of hospitalization due to psychosis were observed in individuals ineligible owing to treatment resistance, tardive dyskinesia, and history of suicide attempts (Table 4). Finally, with more ineligibility criteria met, larger risks of hospitalization due to psychosis were observed in both countries (eResults and eTables 2 and 3 in the Supplement).

**Table 4.** Risk of Hospitalization Due to Psychosis Within 12 Months of Follow-up for the Population of Individuals Remaining After Applying Each Specific Exclusion Criterion Separately Compared With Individuals Who Did Not Meet Any Exclusion Criteria (Eligible Group)

Eligibility	Finnish cohort					Swedish cohort				
	No. of individuals	Relapsed, No. (%)	Time to event/censoring, mean (SD), d	HR (95% CI)^a^	*P* value	No. of individuals	Relapsed, No. (%)	Time to event/censoring, mean (SD), d	HR (95% CI)^a^	*P* value
**Age <18 and >65 y**										
Eligible	3580	615 (17.2)	278 (130)	0.71 (0.61-0.83)	<.001	1619	240 (14.8)	273 (127)	1.04 (0.78-1.38)	.80
Ineligible	1795	212 (11.8)	267 (133)			403	60 (14.9)	264 (131)		
**Substance use**										
Eligible	3580	615 (17.2)	278 (130)	1.43 (1.29-1.59)	<.001	1619	240 (14.8)	273 (127)	1.88 (1.61-2.21)	<.001
Ineligible	3808	801 (21.0)	228 (143)			1828	430 (23.5)	224 (140)		
**Suicide attempt**										
Eligible	3580	615 (17.2)	278 (130)	1.61 (1.42-1.83)	<.001	1619	240 (14.8)	273 (127)	2.13 (1.79-2.54)	<.001
Ineligible	1690	395 (23.4)	225 (144)			1032	270 (26.2)	219 (141)		
**Treatment resistance**										
Eligible	3580	615 (17.2)	278 (130)	1.71 (1.52-1.93)	<.001	1619	240 (14.8)	273 (127)	2.31 (1.87-2.85)	<.001
Ineligible	1805	476 (26.4)	242 (143)			450	134 (29.8)	233 (141)		
**Serious somatic disease, broader definition**										
Eligible	3580	615 (17.2)	278 (130)	1.09 (0.99-1.20)	.09	1619	240 (14.8)	273 (127)	1.53 (1.31-1.77)	<.001
Ineligible	7202	1247 (17.3)	252 (138)			2866	586 (20.5)	243 (139)		
**Serious somatic disease, narrower definition**										
Eligible	3580	615 (17.2)	278 (130)	1.10 (0.99-1.22)	.08	1619	240 (14.8)	273 (127)	1.58 (1.34-1.86)	<.001
Ineligible	5087	877 (17.2)	247 (139)			1747	361 (20.7)	236 (138)		
**Mood stabilizer/antidepressant concomitant use**										
Eligible	3580	615 (17.2)	278 (130)	1.10 (1.01-1.22)	.03	1619	240 (14.8)	273 (127)	1.51 (1.30-1.75)	<.001
Ineligible	7983	1456 (18.2)	262 (135)			3281	682 (20.8)	251 (136)		
**Intellectual disability**										
Eligible	3580	615 (17.2)	278 (130)	0.98 (0.79-1.21)	.83	1619	240 (14.8)	273 (127)	1.19 (0.87-1.62)	.28
Ineligible	622	102 (16.4)	269 (133)			282	47 (16.7)	257 (133)		
**Tardive dyskinesia**										
Eligible	3580	615 (17.2)	278 (130)	1.77 (0.95-3.31)	.07	1619	240 (14.8)	273 (127)	2.13 (1.36-3.32)	<.001
Ineligible	40	10 (25.0)	211 (137)			75	21 (28.0)	235 (129)		
**Pregnant or breastfeeding women**										
Eligible	3580	615 (17.2)	278 (130)	0.87 (0.55-1.37)	.55	1619	240 (14.8)	273 (127)	1.29 (0.53-3.13)	.57
Ineligible	143	19 (13.3)	240 (145)			30	5 (16.7)	237 (148)		

Abbreviation: HR, hazard ratio.

^a^ An HR >1 means higher risk in the ineligible group.

Sensitivity analysis in the cohort of clozapine users uncovered similar proportions of individuals being ineligible (5806 [81.6%] in the Finnish cohort and 1346 [80.2%] in the Swedish cohort) for RCTs as in the main analyses (eTable 4 in the Supplement). Results from the second sensitivity analysis were also similar to the primary analysis: of 4727 individuals treated in outpatient care only, 3508 (74.2%) were ineligible for RCT participation (eTable 5 in the Supplement).

## Discussion

In this study, we applied typical inclusion and exclusion criteria of RCTs to the real-world populations of individuals with schizophrenia in Finnish and Swedish national registries. We found that almost 80% of individuals with schizophrenia would be ineligible to participate in typical RCTs and are therefore not represented in them. The most frequent reasons for ineligibility observed in the 2 cohorts were serious somatic comorbidities and concomitant use of mood stabilizers or antidepressants, followed by history of substance use and risk of suicide. Furthermore, we found that RCT-ineligible real-world individuals had, on average, a moderately higher risk for rehospitalization due to psychosis while receiving maintenance treatment with antipsychotics. This increased risk was observed in several subpopulations, ie, individuals ineligible for specific reasons such as substance use, risk of suicide, treatment resistance, or tardive dyskinesia. Moreover, ineligible individuals appeared to have a higher burden of psychiatric and somatic comorbidities, as indicated by increased psychiatric and any-reason hospitalization rates.

We envision the following implications of our findings. Because we showed that the majority of individuals with schizophrenia are not represented by typical RCTs and that clinical outcomes can differ between eligible and ineligible individuals, targeted RCTs, subgroup analyses of RCTs with broader inclusion criteria, and observational cohorts focusing on underrepresented subpopulations are warranted. To date, only a few RCTs have been conducted in specific patient groups.^13,14,15^ Additionally, because approximately 50% of ineligible individuals met somatic comorbidities exclusion criteria in our study, risks of adverse effects and their potential serious consequences as well as the risk of clinically significant pharmacological interactions could be higher in real-world populations than in RCTs. This may require clinical attention and further research after pivotal RCTs and drug market approval.^16^ The latter could exploit the potential offered by electronic health records for screening and recruiting trial participants and therefore enrich their real-world representativeness. It also underlines the importance of aftermarket/postapproval studies (ie, phase 4 studies) requested by regulators and conducted by pharmaceutical companies to particularly investigate the safety of new treatments in broader populations. Furthermore, we found that choices of antipsychotics were somewhat different between ineligible vs eligible individuals. In previous real-world studies using within-individual designs minimizing selection bias, LAI antipsychotics were associated with lowered risk of rehospitalization whereas quetiapine often was not, compared with no use of antipsychotics.^17,18^ Ineligible individuals were less likely to use LAI antipsychotics and more likely to use quetiapine than eligible individuals. Reasons for these differences are not fully clear from our data; however, it is possible that LAI antipsychotics are avoided as those are slower to taper, eg, in persons with high risk of extrapyramidal symptoms, or active substance use, which increases the risk of interactive effects leading possibly to respiratory depression or seizures. Quetiapine may be prescribed more often for subgroups presenting more affective symptoms (eg, with suicidal ideation) or for tardive dyskinesia.^19^ This also describes a fundamental difference between real-world studies (such as the present study) and RCTs: in the real world, treatments are chosen by clinicians by their best judgment and following clinical care guidelines, while in RCTs the treatment is preset by the design. Therefore, to further elucidate antipsychotic use and effectiveness in practice, in addition to typical RCTs, which are important to examine whether a drug works in principle in selection bias–free conditions (efficacy), pragmatic trials (such as STAR*D^20^ and CATIE^21^) and observational studies (such as the SOHO study^22,23^) may in future be of benefit.^24^ These are performed on less selected populations and resemble clinical practice more closely than typical RCTs. Finally, our results provide estimates for risks of rehospitalization for schizophrenia in different patient populations, which could be used to inform individuals and clinicians about the expected outcome on antipsychotics within 1 year. Of note, individuals with a previous history of substance use, suicide attempt, or clozapine use (as a proxy for treatment resistance) had only a moderately higher risk of rehospitalization for acute psychosis. In this context, it needs to be considered that these estimates only apply to individuals with schizophrenia already stable taking medication for 12 weeks before the start of the 1-year observation period. For some individuals in these subpopulations, it might be difficult to reach this level of stability. Nevertheless, the observed differences in rehospitalization rates between subpopulations call for more specific epidemiological studies on expected absolute risks and predictors of relapse and rehospitalization.

### Limitations

Our analysis is somewhat limited because our selection of individuals eligible for RCTs matches the population in actual RCTs only to a certain extent for different reasons. (1) Participation in a trial requires participants not only to meet eligibility criteria but also to be willing to participate in a trial; the latter might be an important driver of outcomes, which we cannot disentangle in the real-world population. This, in addition to other factors such as dropouts, might explain the difference in rehospitalization rates on antipsychotic maintenance therapy between RCTs (4% at 7-12 [median, 9] months)^10^ and the real world (here 14% at 9 months in eligible individuals). (2) The selection criteria used for our analysis are typical for a specific, relatively common type of RCTs (ie, relapse prevention of schizophrenia with antipsychotics) and specific real-world samples (Finland and Sweden). Therefore, our results may not be directly generalizable to other types of RCTs or to countries with different health care systems or resources. Although the main results represent only individuals previously treated in inpatient care due to schizophrenia spectrum disorders, additional analyses in the Swedish outpatient cohort showed similar results, allaying concerns about bias resulting from cohort and statistical method selections. (3) Furthermore, inclusion and exclusion criteria vary between RCTs (eAppendix 1B in the Supplement). Some RCTs apply more relaxed criteria than the ones we used, eg, by allowing the participation of individuals with psychiatric or somatic comorbidities, with stable concomitant antidepressant or mood-stabilizing medications, history of suicide attempts (without active suicidal thoughts or behaviors), or substance use (when inactive at the time or when criteria for dependence are not met). Of note, the data from real-world cohorts do not allow one to apply all eligibility criteria exactly as in RCTs because only diagnoses and not clinical ratings are available, and some symptoms are often underreported in diagnostic data (eg, suicidality and substance use). However, previous research has shown that register-based inpatient diagnoses of schizophrenia are valid.^25^ Consequently, while our estimates refer to standard RCT with rather strict criteria, possibly other RCTs represent more than the 20% of real-world patients. Nonetheless, our results highlight that there is considerable heterogeneity in real-world individuals, which is not addressed by most standard RCTs.

## Conclusions

In conclusion, based on comprehensive main and sensitivity analyses leveraging sizeable nationwide cohorts and in line with hypotheses put forward before but backed with less solid evidence,^26,27,28,29,30,31^ only a minority (about one-fifth) of real-world individuals with schizophrenia may be eligible for typical RCTs and their clinical outcomes were estimated to differ from ineligible individuals. However, because we did not investigate relative treatment effects (eAppendix 1A in the Supplement) and because the observed differences in absolute risks for clinical outcomes were not extreme, we emphasize that there are no major indications from our research that overall RCT results on efficacy and safety of antipsychotics would not apply to ineligible individuals. Nevertheless, our results indicate that specific subgroups among the majority of real-world individuals ineligible for RCTs can have a different course of illness, which also means they might experience differential treatment benefits. Therefore, in line with previous literature,^28,30,32,33,34,35,36^ future studies focusing on specific subpopulations, pragmatic trials to investigate treatment strategies, and well-designed observational studies are needed to investigate and improve the outcomes of the many individuals afflicted by schizophrenia and currently underrepresented in research settings.

## Notes

Supplement. eMethods. eAppendix 1A. Additional details about methods eAppendix 1B. Eligibility criteria of RCTs on relapse prevention of schizophrenia with antipsychotics eAppendix 2. The RECORD statement – checklist of items, extended from the STROBE statement, that should be reported in observational studies using routinely collected health data eResults. eTable 1. Basic characteristics of the Finnish and Swedish cohorts and overlap between specific exclusion criterion with each other eTable 2. Risk of rehospitalisation within 12 months when stratified by the number of exclusion criteria met in comparison with eligible individuals eTable 3. Frequencies of mood stabiliser (MS) or antidepressant (AD) use among persons with schizophrenia or schizoaffective disorder eTable 4. Additional analyses on eligibility of clozapine users eTable 5. Additional analyses on individuals only treated in outpatient care eFigure 1. Proportion of specific antipsychotics used by individuals eligible vs ineligible for randomised controlled trials in the Finnish and Swedish cohorts eFigure 2. Proportion of specific antipsychotics used within individuals ineligible due to specific reasons in the Finnish cohort eFigure 3. Proportion of specific antipsychotics used within individuals ineligible due to specific reasons in the Swedish cohort eReferences.
