# Supplementary material for: Representation and Outcomes of Individuals With Schizophrenia Seen in Everyday Practice Who Are Ineligible for Randomized Clinical Trials
Source: JAMA Psychiatry. 2022 Jan 26;79(3):1–9. doi: 10.1001/jamapsychiatry.2021.3990 (PMC8792792; doi:10.1001/jamapsychiatry.2021.3990)
Supplement: Supplement. — eMethods. eAppendix 1A. Additional details about methods eAppendix 1B. Eligibility criteria of RCTs on relapse prevention of schizophrenia with antipsychotics eAppendix 2. The RECORD statement – checklist of items, extended from the STROBE statement, that should be reported in observational studies using routinely collected health data eResults. eTable 1. Basic characteristics of the Finnish and Swedish cohorts and overlap between specific exclusion criterion with each other eTable 2. Risk of rehospitalisation within 12 months when stratified by the number of exclusion criteria met in comparison with eligible individuals eTable 3. Frequencies of mood stabiliser (MS) or antidepressant (AD) use among persons with schizophrenia or schizoaffective disorder eTable 4. Additional analyses on eligibility of clozapine users eTable 5. Additional analyses on individuals only treated in outpatient care eFigure 1. Proportion of specific antipsychotics used by individuals eligible vs ineligible for randomised controlled trials in the Finnish and Swedish cohorts eFigure 2. Proportion of specific antipsychotics used within individuals ineligible due to specific reasons in the Finnish cohort eFigure 3. Proportion of specific antipsychotics used within individuals ineligible due to specific reasons in the Swedish cohort eReferences. [file jamapsychiatry-e213990-s001.pdf]

# Supplemental Online Content

Taipale H, Schneider-Thoma J, Pinzón-Espinosa J, et al. Representation and outcomes of individuals with schizophrenia seen in everyday practice who are ineligible for randomized clinical trials. *JAMA Psychiatry*. Published online January 26, 2022. doi:10.1001/jamapsychiatry.2021.3990

**eMethods.**

**eAppendix 1A.** Additional details about methods

**eAppendix 1B.** Eligibility criteria of RCTs on relapse prevention of schizophrenia with antipsychotics

**eAppendix 2.** The RECORD statement – checklist of items, extended from the STROBE statement, that should be reported in observational studies using routinely collected health data

**eResults.**

**eTable 1.** Basic characteristics of the Finnish and Swedish cohorts and overlap between specific exclusion criterion with each other

**eTable 2.** Risk of rehospitalisation within 12 months when stratified by the number of exclusion criteria met in comparison with eligible individuals

**eTable 3.** Frequencies of mood stabiliser (MS) or antidepressant (AD) use among persons with schizophrenia or schizoaffective disorder

**eTable 4.** Additional analyses on eligibility of clozapine users

**eTable 5.** Additional analyses on individuals only treated in outpatient care

**eFigure 1.** Proportion of specific antipsychotics used by individuals eligible vs ineligible for randomised controlled trials in the Finnish and Swedish cohorts

**eFigure 2.** Proportion of specific antipsychotics used within individuals ineligible due to specific reasons in the Finnish cohort

**eFigure 3.** Proportion of specific antipsychotics used within individuals ineligible due to specific reasons in the Swedish cohort

**eReferences.**

This supplemental material has been provided by the authors to give readers additional information about their work.

© 2022 Taipale H et al. *JAMA Psychiatry*.

© 2022 Taipale H et al. *JAMA Psychiatry*.

## eMethods

Our expectation was that a minority of individuals with schizophrenia in the real world are eligible for RCTs. We also expected that outcomes of individuals ineligible for RCTs are worse than of RCT-eligible patients.

### eAppendix 1A: Additional details about methods

We identified standard inclusion and exclusion criteria from 53 typical RCTs of relapse prevention with second-generation antipsychotic drugs in schizophrenia.[1] All disorders and disease codes noted below correspond to the International Classification of Diseases (ICD)-10.[2]

<u>Standard RCT inclusion criteria were:</u>

1) A diagnosis of schizophrenia (F20) or schizoaffective disorder (F25).
2) Clinical stability for at least 12 weeks on antipsychotic treatment (operationalized as continuous use – see 'Prescriptions to Drug Use Periods' (PRE2DUP) method below - and no hospitalisation for 12 weeks).

<u>Standard RCT exclusion criteria were:</u>

1) Age less than 18 years.
2) Age more than 65 years.
3) History of substance abuse (F10-F19, excluding F17 (nicotine), diagnosed ever before follow-up).
4) Risk of suicide (suicide attempt X60-84 (only intentional ones), diagnosed ever before follow-up).
5) Treatment resistance, defined as clozapine or electroconvulsive therapy (ECT) use, reported ever before follow-up.
6) Serious somatic disease, defined in two ways:
   a. Broad: All being treated for A00-N99, excluding F00-F99, during 2 years before start of follow-up.
   b. Narrow: All diagnosed ever before follow-up with the following specific diseases: Neuroleptic malignant syndrome (G21.0), Central Nervous System disorders (Any neurological disease G00-G99), Head injuries (S00-S09), Cardiac disorders (Ischemic heart diseases I20-I25, Other forms of heart disease I30-I52, Cerebrovascular diseases I60-I69, Diseases of arteries, arterioles, and capillaries I70-I79), Abnormal white blood cell count (Agranulocytosis D70).
7) Mood stabilizer or antidepressant use (no use at baseline and censor to initiation of carbamazepine, valproic acid, lamotrigine, lithium, or any antidepressant (Anatomical Therapeutic Chemical (ATC) code N06A)).
8) Intellectual disability/Mental retardation (F70-F79, diagnosed ever before follow-up).
9) Tardive dyskinesia (G24.0, diagnosed ever before follow-up).
10) Pregnant or breastfeeding women (any person with O-code one year before and after is removed from analyses).

### Details about the two nationwide cohorts used in this study

<u>Finnish cohort:</u> This was a prevalent cohort, where participants were identified on the basis of having inpatient care diagnoses of schizophrenia or schizoaffective disorder (F20, F25, referred to onwards as 'schizophrenia') during the years 1972-2014 and followed-up between 2005 and 2017. To enhance comparability with the Swedish cohort, we included individuals in their working age (16-65 years) at cohort entry in 2005 or at first diagnoses for those diagnosed between 2005-2014. Further details of the cohort have been described previously.[3]

<u>Swedish cohort:</u> This was a prevalent cohort identified from the National Patient Register (inpatient, specialised outpatient care) and the MiDAS (Micro-Data for Analysis of the Social Insurance System) register (sickness absence, disability pension) as having been treated due to schizophrenia or schizoaffective disorder (F20, F25, referred to onwards as 'schizophrenia') in 2005-2013 at age of 16-65 years, and followed-up between 2006 and 2016. For comparability purposes with the Finnish cohort, the cohort was restricted to those who had at some point before or during the follow-up received inpatient care due to schizophrenia (F20, F25). In addition to the patient register and prescribed drug register data, we also used data on disability pensions (from MiDAS).

© 2022 Taipale H et al. *JAMA Psychiatry.*

Disability pension data was used in the sensitivity analyses and used as a measure of severe functional impartment. The cohort has been extensively described previously.[4]

Of note, data on involuntary treatment was not available for either of the cohorts.

**Separate cohorts formed as sensitivity analyses**

Clozapine users: For comparison purposes, the same criteria as presented above (except treatment resistance) were also applied to clozapine users and their eligibility vs. ineligibility were assessed the same way as for the main analyses and cohorts.

Outpatient care-treated patients: Individuals who were only treated in outpatient care before start of follow-up were identified from Swedish registers (specialised outpatient care, sickness absence, disability pension). The same criteria as presented above were also applied to this outpatient cohort and their eligibility vs. ineligibility were assessed the same way as for the main analyses and cohorts.

**Note to statistical approach and interpretation**

Regarding the analyses of differences in key outcomes, we did not adjust for any variables in our analyses but presented descriptive results reflecting the numbers in the RW. We followed this approach because our aim was not to make causal statements about differential effects of treatments between populations, in which case confounding would indeed be an issue. Thus, from the differences in risks of rehospitalisations presented we cannot infer that antipsychotics are less effective in the overall-excluded population or specific subpopulations. This research question falls outside our scope and would require adjustment for confounding factors that are not measured in the registries we used,[5] such as baseline illness severity or duration of untreated psychoses.

Concerning relative treatment effects, the most recent meta-analysis of antipsychotics versus placebo in RCTs found an average relative risk of rehospitalisation for psychosis of 0.36 in RCT.[6] In contrast, the hazard ratio of psychiatric rehospitalisation (or death) of antipsychotic users versus discontinuation non-users was 0.53 in the Finnish RW-data.[7] This indicates that treatment effects may differ between RCT- and RW-populations, but one can also argue that they point to the same direction and are in the same ballpark.

**Patient and public involvement statement**

As this study was specifically aimed to represent individuals with schizophrenia in the real-world, we believe our findings are valuable to them. As no intervention was applied and the study mostly concerned statistical considerations, individuals were not involved in the set-up. An experienced expert (RC) was asked to provide feedback on our paper and his comments were processed. The experienced expert stated that the inclusiveness of all sorts of individuals with a history of psychosis in research is fundamental to improving their quality of life. He asserted our findings are a pivotal first step in quantifying the degree to which many individuals with schizophrenia are underrepresented in clinical trials and that the treatment response of some individuals may be different from others. He believed our research might contribute to more personalised and inclusive clinical trial designs for individuals vulnerable to develop psychosis.

**Data Sharing**

The datasets analysed in this study are not publicly available due to participant privacy and security concerns. Researchers can apply for access to these data from the register holders: for Finnish data with the Social Insurance Institution of Finland (Prescription Register), the Finnish National Institute for Health and Welfare (Hospital Discharge Register), and Statistics Finland (Causes of Death Register); for Swedish data with National Board of Health and Welfare (National Patient Register, Prescribed Drug Register), Statistics Sweden (causes of

© 2022 Taipale H et al. *JAMA Psychiatry*.

Death and sociodemographic data in LiSA register), and the Swedish Social Insurance Agency (MiDAS register).

© 2022 Taipale H et al. *JAMA Psychiatry*.

## eAppendix 1B: Eligibility criteria of RCTs on relapse prevention of schizophrenia with antipsychotics

Below, in alphabetical order, we present the eligibility criteria of RCTs that we screened to identify and operationalise key exclusion criteria of typical RCTs. Those RCTs were included in a recent systematic review on relapse-prevention of schizophrenia with antipsychotics.[1] Here, we focused on modern studies, i.e. studies examining second-generation antipsychotics.

Of note, many eligibility criteria were available from journal publications only, which appear to focus on the criteria deemed most relevant to eachspecific study. As it can be seen from the trials for which eligibility criteria from clinical study reports (CSRs) were available, full listings of exclusion criteria are too detailed to be reported in the word-count limited manuscripts submitted to journals. As a consequence, we cannot be certain whether a criterion was not applied or rather not mentioned.

Moreover, as stated in the limitations section of our manuscript, eligibility criteria vary between trials and the accessible data from the Scandinavian patient registries (mainly diagnoses and prescriptions) do not allow to apply these criteria to the real-world-population exactly in the same way as done in RCTs. Thus, we needed to define a set of typical exclusion criteria and operationalise them in a way that could be applied to the patient registries. This was particularly relevant concerning psychiatric and somatic comorbidities and concomitant medications:, as oftenparticipants are excluded from RCTs when they have another "active" psychiatric comorbidity. However, the diagnoses in the registries do not specify whether the disorder is currently active or not. Therefore, to approximate the number of patients with clinically relevant active psychiatric comorbidities, we defined them as those patients using the two other major classes of psychiatric medication, i.e., antidepressants and mood stabilisers. Thereby, we also chose a rather strict approach concerning the use of the major concomitant psychiatric medications, which is handled heterogeneously in RCTs, with some RCTs prohibiting any use whatosever and others prohibiting specific medication or prohibiting initiation or change of concomitant medication only. RCTs also vary concerning the exclusion of somatic comorbidities;often there is a lack of information , as papers only state that participants with clinically relevant somatic comorbidities were excluded. This, we addressed by applying two exclusion criteria concerning somatic comorbidities: a broad criterion excluding all patients with any diagnosis other than F-diagnoses (i.e., psychiatric diagnoses) according to ICD-10 in the past 2 years, and a narrow criterion excluding patients ever diagnosed with the specific diagnoses explicitly mentioned in the exclusion criteria presented below (see eMethods above for a listing of the specific diagnoses).

Overall, as mentioned in discussion section of the manuscript, our eligibility criteria are derived from rather strict RCTs and the estimate of 80% of real-world patients excluded may be considered an upper estimate.

### Studies screened for inclusion and exclusion criteria

#### Arato 2002

Inclusion criteria

Inpatients, aged 18 years or older, with chronic, stable schizophrenia DSM-III-R who had been hospitalized for at least 2 months and had scores of =<5 (markedly ill) on the Clinical Global Impression of Severity scale (CGI-S) were eligible. Previous treatment with ziprasidone was prohibited.

Exclusion criteria

Patients were excluded if they had a recent acute exacerbation of schizophrenia, a score of >= 5 on items P7 (hostility) or G8 (uncooperativeness) of the Positive and Negative Syndrome Scale (PANSS), or displayed a significant risk of suicide. Other exclusion criteria included treatment resistance (defined as lack of therapeutic response to a conventional antipsychotic during an acute exacerbation on at least two occasions in the previous 2 years) and substance abuse or dependence in the previous 3 months. Patients were also excluded if they had been treated with: depot neuroleptics, unless the last injection had been at least one treatment cycle before entry, an investigational drug within the previous 4 weeks, fluoxetine in the previous 5 weeks, monoamine oxidase inhibitors in the previous 2 weeks, or antidepressants or lithium in the previous week. Women of childbearing potential were eligible if they were using reliable contraception. Pregnant or breast-feeding women were excluded.

**Arvanitis 1993**

Inclusion criteria
a) chronic or subchronic schizophrenia according to DSM-III- criteria for any of the following subtypes: disorganized, catatonic, paranoid, residual, or undifferentiated; (b) no concurrent Axis I DSM-III-R diagnoses such as alcohol or psychoactive substance dependence not in full remission, concomitant organic mental disorder, or mental retardation; (c) full or partial remission according to DSM-III-R criteria upon entry into Segment A. The following qualification criteria were met at entry to the trial and at completion of Segment A for the patient to have been randomized to treatment: (d)18 -item BPRS (0 to 6 point system) positive symptom items rated 3 (moderate) for the following: Item 4: Conceptual disorganization; Item 11: Suspiciousness; Item 12: Hallucinatory behavior; Item 15: Unusual thought content; (e) CGI Severity of Illness item assessment 4 (moderately ill)

Exclusion criteria
Any significant clinical disorder, electrocardiogram (ECG), or laboratory finding which in the opinion of the investigator made the patient unsuitable for receiving an investigational drug

**Bai 2006**

Inclusion criteria
Patients enrolled in this study were 18 to 65 years old and had been given a DSM-IV diagnosis of schizophrenia disorder. All were symptomatically stable, receiving (PANSS)" total scores of less than 80 at the screening visit and at baseline. Each of the following PANSS parameters had scores of less than 4 as well: conceptual disorganization, hallucinatory behavior, suspiciousness, and unusual thought content. Differences between Clinical Global Impressions scale (CGI)23 scores at the screening visit and at baseline were less than 1. Inclusion criteria were (1) previous treatment with oral risperidone for at least 3 months prior to the screening visit and (2) otherwise good health, based on the results of a physical examination and blood biochemistry and hematology tests performed at the screening visit and on a medical history.

Exclusion criteria
These were (1) a history of neuroleptic malignant syndrome, documented organic disease of the central nervous system, or current seizure disorder; (2) current risk of violent behavior against other individuals; and (3) current suicidal ideation or suicidal ideation during the 6 months preceding screening.

**Beasley 2003**

Inclusion criteria
Entry requirements: (1) minimal symptoms defined as a BPRS score of no more than 36 at baseline (with relatively little fluc- tuation for >=4 weeks prior to study entry); (2) outpatient status; (3) Global Assessment of Functioning score of >=40; (4) current maintenance on an antipsychotic agent(s) other than clozapine at either >=300 mg/d chlorpromazine equiv- alent for oral agents or >=25 mg every 2 weeks of fluphenazine decanoate equivalent for injectable agents; and (5) lack of specific positive symptoms, as measured by a score of <=4 on the BPRS positive items (scored 1–7) of conceptual disorganization, suspiciousness, hallucinatory behavior, and unusual thought content. Patients who did not meet relapse criteria were eligible for double-blind maintenance phase

Exclusion criteria
Patients were excluded from the study for any of the following reasons: investigators and their immediate families, defined as the investigator's spouse, parent, child, grandparent, or grandchild; persons who had previously participated in this study or any other study investigating the maintenance or relapse prevention capabilities of olanzapine; participation in a clinical trial of an investigational drug within 1 month (30 days) prior to study entre (Visit 1); female patients who were either pregnant or nursing.

© 2022 Taipale H et al. *JAMA Psychiatry*.

### 9.3.2. Exclusion Criteria

Patients were excluded from the study for any of the following reasons:

[10] Investigators and their immediate families, defined as the investigator's spouse, parent, child, grandparent, or grandchild.

[11] Persons who had previously participated in this study or any other study investigating the maintenance or relapse prevention capabilities of olanzapine.

[12] Participation in a clinical trial of an investigational drug within 1 month (30 days) prior to study entry (Visit 1).

[13] Female patients who were either pregnant or nursing.

[14] Serious, unstable illnesses including hepatic, renal, gastroenterologic, respiratory, cardiovascular (including ischemic heart disease), endocrinologic, neurologic, immunologic, or hematologic disease such that death was anticipated within 1 year or intensive care unit hospitalization for the disease was anticipated within 6 months.

[15] Uncorrected hypothyroidism or hyperthyroidism.

[16] Narrow-angle glaucoma.

[17] One or more seizures without a clear and resolved etiology. Note: the site must have contacted the sponsor prior to entering a patient who had experienced any seizure.

[18] Current agranulocytosis (absolute neutrophil count <500 $mm^3$).

[19] Current jaundice, positive hepatitis B surface antigen (HBsAg) or positive IgM fraction of the hepatitis B core antibody (anti-HBc[IgM]). Note: Positive total hepatitis B core antibody (anti-HBc) and positive total hepatitis C virus antibody (anti-HCVab) was not exclusionary.

[20] History of allergic reaction to olanzapine or discontinuation of olanzapine due to an adverse event.

[21] DSM-IV substance (except nicotine and caffeine) dependence within the past 2 months in the clinical judgment of the investigator

[22] Treatment with clozapine within 4 weeks of Visit 1.

[23] Treatment with reversible or nonreversible monoamine oxidase inhibitor, guanethidine, or guanadrel within 1 week prior to Visit 2.

[24] Treatment with remoxipride within 6 months (180 days) prior to Visit 2.

[25] Any other medication with primarily central nervous system activity, other than specified in Section 9.4.6.

© 2022 Taipale H et al. *JAMA Psychiatry*.

**Berwaerts 2015**

Inclusion criteria
Patients (men and women aged 18-70 years, inclusive) diagnosed with schizophrenia (by DSM-IV-TR criteria) for at least 1 year before screening and a PANSS total score lower than 120 at screening and baseline were enrolled. Patients symptomatically stable on other LAI antipsychotic treatments were eligible. A stable place of residence for the previous 3 months before screening was mandatory. Patients with schizophrenia for more than 1 year A total score in the PANSS < 120. Signed informed consent
. Women must not be pregnant, breastfeeding, and if capable of pregnancy must practice an effective method of birth control  Men must agree to use a double-barrier method of birth control  Be medically stable on the basis of clinical laboratory tests, physical examination, medical history, vital signs, and electrocardiogram (ECG).

Exclusion criteria
Major exclusion criteria were the following: primary, active DSM-IV diagnosis other than schizophrenia; significant risk of suicidal behavior; history of substance dependence within 6 months before screening; involuntary status in a psychiatric hospital at screening; or history of neuroleptic malignant syndrome, tardive dyskinesia, or any malignant neoplasm in the previous 5 years except basal cell carcinoma. A diagnosis other than schizophrenia, e.g., dissociative disorder, bipolar disorder, major depressive disorder, schizoaffective disorder,  schizophreniform disorder, autistic disorder, primary substance-induced psychotic disorder, dementia-related psychosis  Relevant history or current presence of any significant or unstable medical condition(s) determined to be clinically significant by the Investigator  (ie, obesity, diabetes, heart disease etc). A diagnosis of substance dependence within 6 months before screening  History of neuroleptic malignant syndrome (NMS) or tardive dyskinesia. Clozapine use in the last 2 months when used for treatment-resistant or treatment-refractory illness. Clinically significant findings in biochemistry, hematology, ECG or urinalysis results  Any other disease or condition that, in the opinion of the investigator, would make participation not in the best interest of the patient or that  could prevent, limit, or confound the protocol-specified assessments.

### 3.2.3. Exclusion Criteria

Subjects were excluded from participation in this study if any of the following key exclusion criteria were met:

- A primary, active DSM-IV-TR Axis I diagnosis other than schizophrenia, eg, dissociative disorder, bipolar disorder, major depressive disorder, schizoaffective disorder, schizophreniform disorder, autistic disorder, primary substance-induced psychotic disorder.
- A DSM-IV-TR diagnosis of active substance dependence within 6 months before screening.
- Attempted suicide within 12 months before screening or were at imminent risk of suicide or violent behavior as clinically assessed by the investigator.
- Involuntarily committed to psychiatric hospitalization at the time of screening.
- Relevant history or current presence of any significant or unstable cardiovascular, respiratory, neurological, renal, hepatic, hematologic, endocrine, morbid obesity (BMI >40 kg/m$^2$), immunologic or other systemic disease, encephalopathic syndrome, mental retardation, risk factors for prolonged QT interval, torsade de pointes or sudden cardiac death.
- Biochemistry, hematology, ECG or urinalysis results that were not within the laboratory's normal reference range and were deemed to be clinically significant by the investigator.
- History or evidence of clinically significant hepatic disease (including aspartate aminotransferase [AST] or alanine aminotransferase [ALT] >2 times the upper limit of normal) at screening.
- History of neuroleptic malignant syndrome (NMS) or tardive dyskinesia or any malignancy within the previous 5 years, with the exception of basal cell carcinomas.

© 2022 Taipale H et al. *JAMA Psychiatry*.

**Buller 2011**

Inclusion criteria
Outpatients with a primary diagnosis of schizophrenia according to DSM-IV-TR™ criteria, who: had been stable for the last 4 weeks, indicated by stable antipsychotic medication (no change in medication and no increase in dose) prior to screening; had a score ≤4 (moderate) on the following PANSS items at screening and baseline: – P2 (conceptual disorganisation) – P3 (hallucinatory behaviour) – P6 (suspiciousness/persecution) – P7 (hostility) – G8 (uncooperativeness) – G9 (unusual thought content); and were ≥18 and ≤55 years of age.

Exclusion criteria
Current Axis I primary psychiatric diagnosis other than schizophrenia; not previously received antipsychotic drugs for schizophrenia; acute exacerbation requiring hospitalisation within the last 3 months; clinically significant extrapyramidal symptoms; clinically significant cardiovascular disease, congestive heart failure, cardiac hypertrophy, arrhythmia or bradycardia; congenital long QT syndrome or a family history of this disease, or known acquired QT interval prolongation; significant ECG abnormalities; hypokalaemia or hypomagnesemia; in concurrent treatment with drugs inhibiting the P450 enzymes system CYP3A.

**Chen 2010**

Inclusion criteria
These included a diagnosis of schizophrenia or non-affective psychosis (schizophreniform disorder, schizoaffective disorder, brief psychotic disorder, or psychosis not otherwise specified) according to the DSM-IV. Experienced (equivalent to royal college or board certified) psychiatrists confirmed the diagnosis according to the Chinese version of the structured clinical interview for DSM-IV. In addition, patients had to be aged 18 to 65, to have received antipsychotic drug treatment continuously for at least 12 months, and to have no history of relapse (defined as any increase in positive symptoms leading to adjustment of drug treatment or readmission to hospital). We ascertained this with information from patients, carers, case managers, clinicians, and clinical records. Patients had to be non-psychotic at study entry, as defined by having below threshold scores on five key psychotic symptoms on the positive and negative syndrome scale as well as free of positive symptoms of psychosis for at least eight weeks. Patients also had to score 2 (borderline or questionable mental illness) or below on the clinical global impressions scale.

Exclusion criteria
These were drug induced psychosis and current treatment with clozapine or depot antipsychotics or with mood stabilising drugs (lithium, valproate, or carbamazepine). Other exclusion criteria included poor adherence to treatment (missing >50% of drug, >50% non-attendance at follow-up clinic visits, or a history of patient initiated discontinuation of treatment), and risk of suicide or violence.

**Chzanowski 2006**

Inclusion criteria
This was a 52-week, multicenter, open-label extension study of aripiprazole vs olanzapine in chronic stable or acutely psychotic patients with schizophrenia. It was an optional extension to a randomized, double-blind, 26-week, placebo-controlled, multicenter clinical trial of aripiprazole 15 mg/day for the treatment of patients with chronic, stabilized schizophrenia, reported previously. In the double-blind phase, protocol criteria for relapse were defined as one or more of: a CGI-I score of ≥5; a PANSS score of≥5 on the items of hostility or uncooperativeness on two successive days; or a ≥20% increase in PANSS Total score. Of patients enrolled in the double-blind, 26-week study, two distinct populations were eligible for inclusion into the extension phase reported in this study: (1) stable patients who had completed the acute phase, and (2) patients who met the protocol criteria for relapse and had completed at least 2 weeks of double-blind therapy.

Exclusion criteria
Based on these criteria, 68 patients who received at least 14 days of therapy did not enter the extension (i.e., discontinued for reasons other than relapse). Willingness to participate in the extension was not a prerequisite for inclusion in the acute study. A small number of patients who met the criteria for the extension phase, both those who relapsed and those who completed, chose not to enter the extension period.

© 2022 Taipale H et al. *JAMA Psychiatry*.

**Chue 2005**

Inclusion criteria
At study entry, patients were to be either inpatients or outpatients aged 18–65 years, have a diagnosis of schizophrenia according to DSM-IV criteria, total PANSS score >50, and no clinically relevant abnormal biochemistry, hematology or urinalysis laboratory values. Patients who remained symptomatically stable as indicated by a stable oral risperidone dose and stable Clinical Global Impressions (CGI) scores for the last 4 weeks of the oral risperidone run-in period were eligible for randomization.

Exclusion criteria
Patients were excluded if they had moderate or severe symptoms of tardive dyskinesia at study entry, a history of neuroleptic malignant syndrome, were known to be unresponsive to risperidone, or required mood stabilizers. Patients were also excluded if they had been treated with clozapine within the last 2 months before screening, with a depot antipsychotic within one treatment cycle of screening, or with an antidepressant within 30 days before the run-in period.

**Citrome 2012**

Inclusion criteria
Key inclusion criteria: age 18–75 years; Diagnostic and Statistical Manual of Mental Disorders, fourth edition criteria for a primary diagnosis of schizophrenia or schizoaffective disorder, as established by a structured diagnostic interview; duration of illness (treated or untreated) of at least 1 year; ‘clinically stable’ (nonacute phase of illness) for at least 8 weeks before the baseline [as defined by Clinical Global Impression- Severity (CGI-S) score of up to 4 (at both Screening and Baseline)]; no change in antipsychotic medications (minor dose adjustments for tolerability purposes were permitted) for at least 6 weeks before screening; no hospitalization for psychiatric illness for at least 8 weeks before screening; and moderate or less (<4) severity rating on the Positive and Negative Syndrome Scale (PANSS) items (at both screening and baseline) of delusions, conceptual disorganization, hallucinations, and unusual thought content

Exclusion criteria
Key exclusion criteria were current clinically significant somatic disorders or abnormal laboratory testing; current clinically significant, or history of, alcohol abuse/alcohol- ism or drug abuse/dependence within the last 6 months; clinically significant suicidal ideation, suicidal behavior, or violent behavior in the past 6 months; body mass index (BMI) less than 18.5 or greater than 40 $kg/m^2$; treatment with risperidone within 6 weeks before baseline; a history of a poor or an inadequate response, or intolerability to risperidone; treatment with electroconvulsive therapy within the last 3 months or likelihood for requirement of electroconvulsive therapy during the study; resistance to antipsychotic treatment or a history of treatment with clozapine for refractory psychosis and/or treatment with clozapine within 4 months of the baseline visit; receiving depot neuroleptics, unless the last injection was at least one treatment cycle before randomization; and participation in a previous lurasidone study. Patients who had been treated with a stable dose of antidepressants or mood stabilizers for at least 1 month before the baseline visit were allowed to continue this treatment during the study

**Cooper 2000b**

Inclusion criteria
Male and female patients aged 18–65 years who met the criteria for chronic schizophrenia DSM III-R, had a score of 3 or more on the clinical global impression (CGI) severity of illness scale, had a history of recurrence within the past 18 months and were currently maintained on antipsychotic medication were included in the trial. They could be either in-patients or out-patients. Women of childbearing potential could be included if they were using a reliable form of contraception.

Exclusion criteria
Exclusion criteria were: significant cardiovascular or electrocardiogram (ECG) abnormality; recent myocardial infarction; renal or hepatic failure; blood dyscrasia; epilepsy; Parkinson’s disease; dementia; head trauma or significant neurological illness; severe hypotension or hypertension; prostatic hypertrophy; urinary retention; narrow-angle glaucoma; chronic respiratory disease; asthma; hypersensitivity to antipsychotics; other significant psychiatric illness; clinically significant abnormal laboratory values; alcohol abuse; suicide risk; pregnancy; lactation; breast neoplasm; prolactin-dependent tumour; significant menstrual irregularity; and hyperprolactinemia. Women of childbearing potential could be included if they were using a reliable form of contraception.

© 2022 Taipale H et al. *JAMA Psychiatry*.

**Csernansky 2002**

Inclusion criteria
Eligibility criteria included an age of 18 to 65 years, a diagnosis of schizophrenia or schizoaffective disorder according to the criteria of the DSM-IV, and inpatient psychiatric hospitalization, daytime psychiatric hospitalization, outpatient crisis management, or short-term treatment in a psychiatric hospital emergency room within the 24 months before study entry. All patients had received a stable dose of antipsychotic medication for at least 30 days before entry, had resided at the same address for at least 30 days before entry, and were judged clinically stable by the principal investigator at each site.

Exclusion criteria
Exclusion criteria included another current DSM-IV Axis I diagnosis, an Axis II diagnosis of border- line personality disorder or antisocial personality disorder, current substance dependence or abuse, clinically significant or unstable medical illness, current treatment with clozapine, a history of refrac- toriness to antipsychotic drugs, and treatment with depot neuro- leptic injections within one treatment cycle before screening. Patients who were allergic to either risperidone or haloperidol and women who were pregnant or nursing were also excluded.

**Daniel 1998**

Inclusion criteria
outpatients with schizophrenia who had been clinically stable on neuroleptic medication for at least 3 months before randomization.

Exclusion criteria
Patients were not included if they had a primary psychiatric diagnosis other than schizophrenia, demonstrated violent or suicidal behavior, or had suicidal ideas, prior psychosurgery, or a clinically significant abnormal neurological examination. Those with a clinically significant concurrent medical problem, a score of 4 (severe) on any Abnormal Involuntary Movement Scale (AIMS) item (NIMH 1l985), or a current diagnosis or recent history (2 months) of substance abuse were also excluded.

**Detke 2014**

Inclusion criteria
Study participants were outpatients, 18 to 65 years old, who met the criteria for schizophrenia based on the DSM-IV or the DSM-IV Text Revision. Patients were required to be considered ‘‘at risk for relapse,’’ defined as having experienced at least 2 episodes of clinical worsening of schizo- phrenia symptoms in the previous 24 months such that the patient was hospitalized or required an increased level of care surrounding the episode. Increased level of care could include the addition of or change to any of the following from a lower level of care: day hospital program; outpatient crisis management; short-term psychiatric treatment in an emergency department; or an addition, increase, or switch of medication. Patients were also required to be sufficiently clinically stable at the time of study entry, defined as no acute hospitalization for psychosis in the 8 weeks before visit 1, a Positive and Negative Syndrome Scale (PANSS) total score of lower than 70 at visits 1 and 2, and a Clinical Global Impressions Severity of Illness Scale (CGI-S) of 4 or lower at visits 1 and 2. Finally, the patient and the treating physician were required to have a desire to change the patient’s therapy due to unsatisfactory clinical response, adverse events, or nonadherence to current anti- psychotic therapy.

Exclusion criteria
Exclusion criteria included previous par- ticipation in studies of olanzapine LAI, treatment resistance to olanzapine, previous withdrawal from olanzapine treat- ment due to clinically significant and/or intolerable adverse events, substance dependence (other than nicotine or caf- feine) within the past 30 days, pregnancy, breast-feeding, or serious or unstable medical illness.

**Durgam 2015c**

Inclusion criteria
To be included, male or female inpatients (18–60 years of age, inclu- sive) were required to have a current DSM-IV-TR diagnosis of schizophrenia (minimum 1 year) and a current psychotic episode b 4 weeks' duration. Patients additionally had a PANSS total score ≥ 70 and ≤120, and a score ≥ 4 (moderately severe) on at least 2 PANSS positive symptoms (delusions, hallucinatory behavior, conceptual disorganization, suspiciousness or persecution)

© 2022 Taipale H et al. *JAMA Psychiatry.*

Exclusion criteria
Patients currently meeting DSM-IV-TR criteria for schizoaffective disorder, schizophreniform disorder, bipolar I and II and known or suspected borderline or antisocial personality disorder or other DSM-IV-TR axis II disorders. Patients in their first episode of Psychosis. Treatment-resistant schizophrenia over the last 2 years. Positive result from the blood alcohol test or from the urine drug screen for any prohibited medication. At imminent risk of injuring self or others or causing significant damage to property. Suicide risk

**Eli Lilly NCT00190749**

Inclusion criteria
This was a 12-week, randomized, double-blind study in patients meeting DSM/-IV-TR criteria for schizophrenia or schizoaffective disorder. Participants were men and women aged 18–65 years with stable psychiatric illness [no hospitalizations for ≥ 3 months; a total score on the Brief Psychiatric Rating Scale (BPRS) ≤ 42 and scores ≤ 4 on each BPRS positive symptom item]. Patients who met interim criteria for psychiatric stability (BPRS total score ≤ 46, each BPRS positive item ≤ 4) were eligible for the baseline clamp procedure. Patients who completed the clamp procedure were randomly assigned to 12 weeks of double-blind, flexible-dose treatment with olanzapine or risperidone (1 : 1).

Exclusion criteria
Treatment with olanzapine, risperidone or depot antipsychotics within 4 weeks of study entry, or with clozapine within 2 years of entry, was exclusionary. Individu- als with a body mass index (BMI) >40 kg/m2 were excluded [because of limitationsof thedual-energy X-ray absorptiometry (DEXA) scanning instrument], as were patients with diabetes [fasting glucose ≥ 7.00 mmol/l, glycated haemoglobin >6.5% (47.55 mmol/mol) or prior diagnosis], severe fasting hypertriglyceridaemia (>4.52 mmol/l) or use of medications known to affect insulin secretion or sensitivity.

**EQUATOR**

Inclusion criteria
The trial included male and female inpatients and outpatients aged 18 to 65 years with a diagnosis of schizophrenia for at least 3 years as defined by the DSM-IV-TR. Patients must have been experiencing an acute exacerbation of psychotic symptoms at screening, as demonstrated by a Positive and Negative Syndrome Scale (PANSS) total score of >80. Patients must have shown response to antipsychotic treatment (other than clozapine) in the previous year, be currently treated with oral or depot antipsychotics (other than clozapine) or have a recent lapse in antipsychotic treatment, and have a history of relapse and/or symptom exacerbation in the absence of antipsy- chotic treatment

Exclusion criteria
Exclusion criteria included a DSM-IV-TR Axis I diagnosis other than schizophrenia, acute depressive symp- toms in the previous 30 days requiring antidepressant therapy, antipsychotic-resistant or refractory schizophrenia, a significant risk of violent behavior or suicide, meeting DSM-IV-TR criteria for substance abuse or dependence in the previous 180 days, or requiring prohibited concomitant therapy during the trial.

**Fleischhacker 2014**

Inclusion criteria
Eligible patients were aged 18–60 years and had a diagnosis of schizophrenia according to DSM-IV-TR criteria for >3 years and a history of symptom exacerbation when not receiving antipsychotic treatment. Patients needed to have been responsive to antipsychotic treatment (other than clozapine) in the past year. Other exclusion criteria included patients who were considered to be treatment resistant/refractory to antipsychotic treatment by history. Patients were also excluded if they failed to respond to clozapine treatment or were responsive to clozapine treatment only.

Exclusion criteria
Key exclusion criteria included a DSM-IV-TR diagnosis other than schizophrenia; uncontrolled thyroid function abnormalities; a history of seizures, neuroleptic malignant syndrome, clinically relevant tardive dyskinesia, or other medical condition that would expose the patient to undue risk or interfere with study assessments. Patients who had been admitted to hospital, including for psychosocial reasons, for 430 days total of the 90 days preceding entry into phase 1 or 2 of the study after screening were excluded. Individuals were also excluded if they met DSM-IV-TR criteria for substance dependence, including alcohol and benzodiazepines but excluding nicotine and caffeine. Additional exclusion criteria were noted in online supplement.

© 2022 Taipale H et al. *JAMA Psychiatry*.

**Fricchione 2010**

Inclusion criteria
This pragmatic, observational, independent study is based on 40 intent-to-treat (ITT) outpatients with DSM IV diagnosis of schizophrenia or schizoaffective or delusional disorder.

Exclusion criteria
Exclusion criteria: disorders or conditions contraindicating either of investigational drug.

**Fu 2015**

Inclusion criteria
Subjects were required to have a lifetime and current diagnosis of schizoaffective disorder according to the Structured Clinical Interview for DSM-IV Axis I Disorders (SCID) (Clinician Version) conducted during screening. Men and women aged 2 18 years with an acute exacerbation of psychotic symptoms 2 4 days and s 4 weeks in duration before screening and willing to accept long-acting injectable treatment were eligible. To ensure recruitment of patients who were experiencing exacerbation ofpsychotic symptoms, subjects had to have a score of 24 (moderate) on 2 3 of the following PANSS items: P1 (delusions), P2 (conceptual dis-organization), P3 (hallucinatory behavior), P4 (excitement), P6 (suspiciousnesslpersecution), P7 (hostility), G4 (tension), G8 (uncooperativeness), or G14 (poor impulse control). Subjects were also required to have prominent mood symp- toms (YMRS and/or HDRS-21 scores 216) at screening.

Exclusion criteria
Key exclusion criteria for this study were as follows: positive urine screen for cocaine, opiates, phenylcyclohexylpiperidine, or amphetamines; meeting DSM-IVcriteria for major depressive disorder, bipolar disorder, or schizophrenia; meeting criteria for any other Axis I diagnosis except substance abuse; having an Axis II diagnosis of mental retardation or borderline personality disorder; meeting the DSM-IV criteria for substance dependence (except for nicotine and caffeine dependence in the 3 months before the screening visit; having attempted suicide within 12 months before the screening visit judgment; being in a first episode of psychosis (no prior history of psychotic symptoms); having received therapy with both mood stabilizers and antidepressants, or having received therapy with mood stabilizers or antidepressants that have been initiated or changed in dose within 30 days prior to screening.

**3.2.3. Exclusion Criteria**

Potential subjects who met any of the following criteria were excluded from participating in this study:

- Had a urine drug screen that was positive for cocaine, opiates, phenylcyclohexylpiperidine (PCP), or amphetamines.
- Met the DSM-IV criteria for major depressive disorder, bipolar disorder, or schizophrenia.
- Met criteria for any other Axis I diagnosis except substance abuse.
- Had an Axis II diagnosis of Mental Retardation or Borderline Personality Disorder.
- Met the DSM-IV criteria for substance dependence (except for nicotine and caffeine dependence) in the 3 months before the screening visit.
- Had attempted suicide within 12 months before the screening visit or were at imminent risk of suicide or violent behavior according to the investigator's clinical judgment.
- Were in their first episode of psychosis (no prior history of psychotic symptoms).

34

© 2022 Taipale H et al. *JAMA Psychiatry*.

- Had a history of receiving electroconvulsive therapy in the 3 months before the screening visit.
- Had a history of hypersensitivity to or intolerance of paliperidone, risperidone, or 20% Intralipid (placebo) or any of their excipients (eg, soybean oil, egg yolks, phospholipids, glycerol).
- Had participated in a clinical investigation or received an experimental therapy within 30 days before the screening visit or had participated in >2 clinical studies within the last 12 months.
- Were previously enrolled in this study.
- Received long-acting antipsychotic medication within 2 injection cycles prior to the screening visit.
- Received therapy with clozapine within 3 months of the screening visit with the exception of low dose clozapine used for treatment of insomnia.
- Had a history of neuroleptic malignant syndrome.
- Had previous history of lack of response to antipsychotic medication. Lack of response was defined by failure to respond to 2 adequate trials of different antipsychotic medications; an adequate trial is defined as a minimum of 4 weeks at the subject's maximum tolerated dose.
- Received therapy with both mood stabilizers and antidepressants; or received therapy with mood stabilizers or antidepressants that has been initiated or changed in dose within 30 days prior to screening.
- Received therapy with carbamazepine.
- Received therapy with monoamine oxidase inhibitors.
- Had a history or presence of circumstances that may increase the risk of the occurrence of torsade de pointes and/or sudden death in association with the use of drugs that prolong the corrected QT (QTc) interval.
- Had a serious and/or unstable neurological disease, including but not limited to Alzheimer's disease, vascular dementia, Parkinson's disease, intracranial lesions, hydrocephalus, significant head trauma, or seizure disorder
- Had a relevant history of or current presence of any significant and/or unstable cardiovascular, respiratory, neurological (including seizures or significant cerebrovascular), renal, hepatic, hematologic, endocrine, immunologic, morbid obesity, or other systemic disease

Refer to the study protocol for a complete list of exclusion criteria.

**Gaebel 2007**

<u>Inclusion criteria</u>
Inclusion criteria were (l) having successfully completed acute treatment (within the 8-week acute trial or with haloperidol in standard routine inpatient care) for schizophrenia (according to ICD-10 F20) in the first illness episode (defined as the first inpatient treatment of the respective symptoms and no former treatment with antipsychotic medication); (2) being aged from 18 to 55 years; (3) being sufficiently proficient in German language; (4) having no involuntary inpatient treatment (at the date of inclusion); and (5) providing written informed consent after explicit information about the study.

<u>Exclusion criteria</u>
Exclusion criteria were (1) pregnancy, (2) contraindication for antipsychotic treatment, (3) mental retardation, (4) organic brain disease, (5) substance dependence, (6) suicidal behavior in previous history, (7) serious physical disease, and (8) participation in other incompatible trials.

© 2022 Taipale H et al. *JAMA Psychiatry.*

**Gaebel 2010**

Inclusion criteria
Symptomatically stable adults aged >18 years were eligible if they met DSM-IV criteria for schizophrenia or schizoaffective disorder, and were candidates for switching therapy because of insufficient symptomatic control, side effects, or patient request. Candidates were considered symptomatically stable when using a stable dose of antipsychotic for >4 weeks (including monotherapy with oral risperidone 6 mg daily, olanzapine 20 mg daily, or a conventional neuroleptic 10 mg haloperidol or its equivalent) and were living in the same residence for >30 days

Exclusion criteria
Patients were excluded if they were previously determined to be nonresponders to risperidone, quetiapine, or >2 antipsychotics despite adequate drug plasma levels (previous nonresponders because of nonadherence were not excluded); had a DSM-IV axis I diagnosis other than schizophrenia or schizoaffective disorder; were treated with antipsychotics other than oral risperidone, olanzapine, or conventional oral neuroleptics or with mood stabilizers or antidepressants and had not received a stable dose for >3 months before study entry; had phenylketonuria or hypersensitivity to risperidone or quetiapine; had drug or alcohol dependence during the preceding 1 month; or were at acute risk or had a history of suicide attempt(s).

**Glick 2005**

Inclusion criteria
Patients with DSM-IV diagnosed schizophrenia or schizoaffective dissorder which required long-term therapy were eligible. No specific inclusion criteria.

Exclusion criteria
No specific exclusion criteria.

**H. Lundbeck 2009**

Inclusion criteria
Inpatients, partially hospitalised patients, or outpatients with a primary diagnosis of schizophrenia according to DSM-IV-TR criteria, who: were ≥18 and ≤65 years of age; had a Clinical Global Impression – Severity of Illness (CGI-S) score ≥4 (moderately ill) at screening and at baseline;had a Positive and Negative Syndrome Scale (PANSS) total score ≥60 at screening and at baseline; had a score of ≤4 on the following PANSS items at screening and at baseline: P2 (conceptual disorganisation), P7 (hostility) and G8 (uncooperativeness); were in the maintenance phase and did not have an acute exacerbation within 8 weeks prior to screening or 4 weeks prior to baseline; did not have their antipsychotic medications changed within 8 weeks prior to screening (agent) or between screening and baseline (agent and dose).

Exclusion criteria
The subject is either at significant risk of suicide, treatment resistant , has experienced an acute exacerbation within 8 weeks prior screening, is unlikely to comply with the protocol, or has a current diagnosis or a history of substance abuse

**Hirsch 2002**

Inclusion criteria
Outpatients aged 16-64 y with a primary diagnosis of chronic or subchronic schizophrenia (DSM-III-R) who required antipsychotic maintainance treatment were entered into the study. Patients with a score of >= 10 on the negative subscale of PANSS and a score of >30 on the GAF scale at both screening and baseline were randomized

Exclusion criteria
Patients were not allowed to enter if they were experiencing an acute exacerbation, had been hospitalised for psychosis during the 12 weeks before screening, or had a score of >= 5 on PANSS item P7 (hostility) or G8 (uncooperativeness). Patients who deteriorated notably betwen baseline and screening, reaching a CGI-I scale score of >=6 were also excluded. Patients were excluded if they had a history of substance abuse or dependence (DSM-III-R) in the preceding 3 months. Urine samples were required to be negative for all illicit drugs, although patients positive for cannabinoids could enter at the discretion of the investigator. Patients were also excluded if they were at significant risk of suicide or homicide, or had a history of any of the following: allergy to any neuroleptic, neuroleptic malignant syndrome, or known resistance to conventional drugs during acute

© 2022 Taipale H et al. *JAMA Psychiatry.*

exacerbation, defined as failure to experience therapeutic response to marketed antipsychotics at least twice in the previous 2 years. Patients were not allowed to enter if they had taken part in a ziprasidone trial or had received an investigational drug within 4 weeks, fluoxetine within 5 weeks, monoamine oxidaseinhibitors within 2 weeks, or antidepressants or lithium within 1 week of the first day of study therapy. Exclusion criteria also included relevant medical illness, epilepsy, neurologic disorders, human immunodeficiency virus seropositivity, serological evidence of hepatitis infection, or clinically significant electrocardiogram (ECG) or laboratory abnormalities.Women who either were unable to conceive or were reliably using contraception and were not pregnant or breast-feeding were allowed to enter the study.

**Hough 2010**

Inclusion criteria
Men and women, aged 18–65 years (inclusive), with a diagnosis of schizophrenia DSM-IV criteria for at least 1 year before screening, and a PANSS total score below 120, at screening and baseline were enrolled. Both symptomatic and stable patients were eligible.

Exclusion criteria
Major exclusion criteria were: primary, active DSM-IV diagnosis other than schizophrenia; significant risk of suicidal or aggressive behavior; history of substance dependence within 3 months before screening; significant medical conditions, or treatment resistance (failure to respond to 2 adequate trials, minimum 4 weeks of antipsychotic medications); use of any 4-week depot antipsychotic within 28 days or risperidone LAI within 5 weeks before screening; use of oral antipsychotics, mood stabilizers, or other prescription or over-the-counter drugs within 2 days before baseline; or involuntary admission to a psychiatric hospital. Women were excluded if pregnant, nursing, or planning to become pregnant.

**3.2.3. Exclusion Criteria**

Potential subjects who met any of the following criteria were excluded from participating in this study:

- A primary, active DSM-IV-TM diagnosis other than schizophrenia;
- A DSM-IV-TM diagnosis of active substance dependence within 3 months before screening (nicotine and caffeine were not exclusionary);
- History of treatment resistance as defined by failure to respond to 2 adequate trials (minimum of 4 weeks at a therapeutic dose) of different antipsychotic medications;
- Relevant history or current presence of any significant or unstable cardiovascular, respiratory, neurological (including seizures or significant cerebrovascular), renal, hepatic, hematologic, endocrine, immunologic or other systemic disease;
- History of any severe pre-existing gastrointestinal narrowing (pathologic or iatrogenic) or inability to swallow the oral study drug whole with the aid of water for subjects requiring oral tolerability testing;
- Biochemistry, hematology or urinalysis results not within the laboratory's normal reference range and deemed to be clinically significant by the investigator;
- History or evidence of clinically significant hepatic disease [including aspartate aminotransferase (AST) or alanine aminotransferase (ALT) >2 times the upper limit of normal (ULN)] at screening;
- History of neuroleptic malignant syndrome (NMS);
- Significant risk of suicidal or violent behavior, as clinically assessed by the investigator;
- History of severe, life-threatening allergic reaction to any drug;

© 2022 Taipale H et al. *JAMA Psychiatry.*

- Known or suspected hypersensitivity or intolerance to risperidone, paliperidone, Intralipid®, or any of their excipients (including egg yolks, soybean oil, phospholipids, and glycerol);
- Exposure to an experimental drug, experimental biologic or experimental medical device within 30 days before screening;
- Previous enrollment in this study or history of having received a previous injection of paliperidone palmitate ;
- History of any malignancy within the previous 5 years, with the exception of basal cell carcinomas;
- Female subjects who were pregnant, breastfeeding or planning to become pregnant during the study phase;
- Treatment with any of the following disallowed therapies:
  - 4-week interval LAI antipsychotic within 28 days before screening
  - RISPERDAL CONSTA LAI antipsychotic within 5 weeks before screening
  - Electroconvulsive therapy with 60 days before screening;
  - Nonselective/irreversible monoamine oxidase inhibitor antidepressants within 4 weeks before screening;
  - Other antidepressants unless at a stable dosage for 30 days before screening (If the dosage has been stable for less than 30 days and the subject does not require the antidepressant, it can be washed out by the baseline visit; if the dosage has been stable for less than 30 days and the subject requires antidepressant treatment, the subject should not be included in this study);
  - Oral antipsychotic within 2 days before baseline (must be washed out by 2 days before baseline);
  - Mood stabilizers within 2 days before baseline (must be washed out by 2 days before baseline), including lithium, valproic acid, topiramate, carbamazepine, and lamotrigine;
  - Other prescription, over-the-counter, or herbal agents with psychoactive properties within 2 days before baseline;
- Employees of the investigator or study center, with direct involvement in the proposed study or other studies under the direction of that investigator or study center, as well as family members of the employees or the investigator;
- Subjects involuntarily committed to psychiatric hospitalization;
- Subjects unable to provide their own consent.

**Ishigooka 2015**

Inclusion criteria

Patients eligible for enrollment in the screening phase were required to be 18 years of age or over and met the DSM-IV-TR criteria for schizophrenia as a diagnosis. Patients were also required to have had a Body Mass Index (BMI) of 18.5–35.0. Patients in the oral stabilization phase (phase 2) were required to meet any of the following: 1) patients who were able to complete conversion to aripiprazole tablet monotherapy within 12

© 2022 Taipale H et al. *JAMA Psychiatry*.

weeks after the start of phase 1, 2) patients receiving aripiprazole monotherapy at time of informed consent, 3) patients considered to be capable of receiv- ing aripiprazole tablet monotherapy and who had not received any antipsychotics for at least 12 weeks, at time of informed consent. In addition, patients were adjusted appropriately to"prohibited concomitant medications" and "restricted concomitant medications" requirements stipulated in this protocol. Patients in the double-blind phase (phase 3) were required to meet the stabilization criteria as stated above.

Exclusion criteria
Patients were excluded if they: had a DSM-IV-TR diagnosis other than schizophrenia; had a complication or a history of diabetes, diabetic ketoacidosis, or diabetic coma; had liver, kidney, heart or hematopoietic organ dysfunction; or were lactating or pregnant. In addition, patients were excluded if they had a complication or a history of polydipsia, Parkinson's disease, tardive dyskinesia, neuroleptic malignant syndrome, rhabdomyolysis, paralytic ileus, alcohol dependence or drug abuse, suicide attempt or self-injury, cerebral vascular disorder, convulsive disorders including epilepsy, organic brain disorder, agranulocytosis or granulocytopenia, or other complications. Patients who had received electroconvulsive therapy within 12 weeks prior to informed consent, who had participated in any other clinical trials within 24 weeks prior to informed consent, for whom clozapine had been ineffective or had responded only to clozapine, and who had been judged by the investigator or subinvestigator to be inappropriate for inclusion in this trial for any other reasons were excluded.

**Kane 2010c**

Inclusion criteria
Participants were 18 to 75 years of age, with a DSM-IV or DSM- IV-TR diagnosis of schizophrenia. Patients were clinically stable, defined as having outpatient status for at least 4 weeks before the first study visit, with a Brief Psychiatric Rating Scale (BPRS) positive symptom subscale score ≤4 (range: 1–7) on each of the following items: conceptual disorganization, suspiciousness, hallucinatory behavior, and unusual thought content. Patients treated previously with a depot antipsychotic were required to have received their last injection at least 2 weeks or one injection interval before entry (4 weeks for injectable risperidone).

Exclusion criteria
Exclusion criteria included significant suicidal or homicidal risk; pregnancy or breastfeeding; acute, serious, or unstable medical conditions; or substance dependence (except nicotine or caffeine) within the past month.

**Kane 2011**

Inclusion criteria
Men and women (2 18 years) with a primary Diagnostic and Statistical Manual ofMental Disorders, Fourth Edition, Text Revision, diagnosis of schizophrenia were eligible. Documented histories of 2 1 prior acute schizophrenia episode during the preceding 3 years and schizophrenia requiring continuous antipsychotic treatment for 21 years preceding screening were required. Patients had to be clinically stable (no antipsychotic dose increase, psychiatric hospital admission, emergency room visit, or psychiatric care increase owing to worsening schizophrenia; no arrest or imprisonment) for >4 weeks before study entry and have a caregiver/ responsible person and access to appropriate supervision during treatment. Women of childbearing age could not be pregnant or breastfeeding and had to be using birth control for >1 month before screening.

Exclusion criteria
Participants were excluded for any of the following reasons: diagnosis of a concurrent Axis I psychiatric disorder; mental retardation, organic brain syndrome, or substance abuse; current acute schizophrenia relapse; PANSS total score > 80 or scores >4 on "unusual thought content," "conceptual disorganization," "hallucinatory behavior," "hostility," or "uncooperativeness" at screening; CGI-S score > 4 (moderately ill); score of 2 on Modified-ISST items 7, 10, or 11; history (within preceding 2 y) or imminent risk of suicide attempt or violence; history ofnoncompliance with antipsy- chotic medication; clozapine use for schizophrenia within preceding 12 weeks; or unstable medical conditions.

**Kane 2012**

Inclusion criteria
Patients were individuals 18-60 years of age requiring chronic antipsychotic treatment who could understand protocol requirements and provide informed written consent. Enrolled subjects had a diagnosis of schizophrenia, as defined by the DSM-IV-TR criteria, for at least 3 years (to exclude first-episode patients) and a history of symptom exacerbation or relapse when not receiving antipsychotic treatment.

Exclusion criteria
Key exclusion criteria included a DSM-IV-TR diagnosis other than schizophrenia, any clinically significant medical or neurologic disorder, and any medically significant abnormal laboratory test or electrocardiogram (ECG) results at screening. Subjects considered refractory to antipsychotic treatment by history or responsive to clozapine treatment were excluded.

### 5.3.2 Exclusion Criteria

Subjects were excluded if they met any of the exclusion criteria listed in Table 5.3.2-1:

**Table 5.3.2-1 Exclusion Criteria**

| | |
|---|---|
| **Sex and Reproductive Status** | |
| 1. | Sexually active males who were not practicing double-barrier birth control or who would not remain abstinent during the trial and for 180 days following the last dose of trial medication, or sexually active females of childbearing potential who were not practicing double-barrier birth control or who would not remain abstinent during the trial and for 150 days following the last dose of trial medication. If employing birth control, 2 of the following precautions must have been used: vasectomy, tubal ligation, vaginal diaphragm, intrauterine device, birth control pill, birth control implant, condom, or sponge with spermicide. |
| 2. | Females who were breast-feeding and/or who had a positive serum pregnancy test result prior to receiving trial medication. |
| **Target Disease** | |
| 3. | Subjects with a current DSM-IV-TR diagnosis other than schizophrenia, including schizoaffective disorder, major depressive disorder, bipolar disorder, delirium, dementia, amnestic or other cognitive disorders. Also, subjects with borderline, paranoid, histrionic, schizotypal, schizoid, or antisocial personality disorder. |
| 4. | Subjects experiencing acute depressive symptoms within the past 30 days, according to the investigator's opinion, that required treatment with an antidepressant. |
| 5. | Subjects with schizophrenia that were considered resistant/refractory to antipsychotic treatment by history. |
| 6. | Subjects with a history of failure to clozapine treatment or response to clozapine treatment only. |
| **Medical History and Concurrent Disease** | |
| 7. | Subjects with a significant risk of violent behavior or a significant risk of committing suicide based on history or investigator's judgment. |
| 8. | Subjects who currently met DSM-IV-TR criteria for substance dependence; including alcohol and benzodiazepines, but excluding caffeine and nicotine. |
| 9. | Subjects with known hypothyroidism or hyperthyroidism (unless condition had been stabilized with medications for at least the past 90 days). |
| 10. | Subjects who had a history or evidence of a medical condition that would have exposed them to an undue risk of a significant AE or interfere with assessments of safety or efficacy during the course of the trial, including but not limited to hepatic, renal, respiratory, cardiovascular, endocrine, neurologic, hematologic, or immunologic disease as determined by the clinical judgment of the investigator. |
| 11. | Subjects with epilepsy or a history of seizures, except for a single childhood febrile seizure, post traumatic, alcohol withdrawal, etc. |
| **Physical and Laboratory Test Findings** | |
| 12. | Subjects with 2 positive drug screens for cocaine prior to the Oral Stabilization Phase. Two positive drug screens for other drugs of abuse were discussed with the medical monitor prior to entry into the Oral Stabilization Phase unless the subject satisfied criteria for dependence, in which case the subject was excluded from the trial. |

© 2022 Taipale H et al. *JAMA Psychiatry.*

| Table 5.3.2-1 | Exclusion Criteria |
|---|---|
| 13. | The following laboratory test, vital sign, and ECG results were exclusionary:<br>1) Platelets ≤ 75,000/$mm^3$<br>2) Hemoglobin ≤ 9 g/dL<br>3) Neutrophils, absolute ≤ 1000/$mm^3$<br>4) Aspartate aminotransferase > 3x upper limit of normal<br>5) Alanine aminotransferase > 3x upper limit of normal<br>6) Creatinine ≥ 2 mg/dL<br>7) Diastolic blood pressure > 105 mmHg<br>8) QTc > 475 msec using either the QTcB (Bazett) or QTcF (Fridericia) corrections on 2 of 3 time points of triplicate ECGs performed (refer to NOTE below)<br><br>NOTE: In addition, subjects were excluded if they had any other abnormal laboratory tests, vital sign results, or ECG findings that, in the investigator's judgment, was medically significant and would impact the safety of the subject or the interpretation of the trial results. Criteria were provided in the protocol appended to the report, to assist investigators in their assessments of results that were potentially medically significant, depending on the subject's medical history and clinical presentation. Abnormal results for laboratory parameters or vital signs were repeated to ensure reproducibility of the abnormality before excluding a subject based on the criteria noted above. The central ECG service provided the corrections for the 3 ECGs done approximately 5 minutes apart (each ECG result reported was derived from the average of the triplicate ECG done at each time point.) Based on the QTcB or QTcF corrections reported by the central service, a subject was excluded if *either* of the corrections exceeded 475 msec for 2 of the 3 time points of triplicate ECGs done. If only one triplicate ECG time point had a corrected QTc greater than 475 msec on either correction factor and this was not reproduced at the other 2 time points, this subject could be included in the trial. |
| **Allergies and Adverse Drug Reactions** | |
| 14. | Subjects who were known to be allergic, intolerant, or unresponsive to prior treatment with aripiprazole or other quinolinones. |
| 15. | Subjects with a history of hypersensitivity to antipsychotic agents. |
| 16. | Subjects who were known to be allergic, intolerant, hypersensitive, or refractory to antipsychotic agents. |
| 17. | Subjects with a history of neuroleptic malignant syndrome or clinically significant tardive dyskinesia at screening. |
| **Prohibited Therapies and/or Medication** | |
| 18. | Subjects likely to require prohibited concomitant therapy during the trial (see Table 5.5.6-1). |
| 19. | Subjects who received CYP2D6 or CYP3A4 inhibitors or CYP3A4 inducers at screening or anticipated use of such agents during the trial (see Table 5.5.6-1 and Section 5.5.6). |
| 20. | Subjects who received any investigational agent in a clinical trial within 30 days prior to screening or who were randomized into a clinical trial with aripiprazole IM depot at any time. NOTE: Subjects who had discontinued at any phase of the trial (the Conversion Phase to the Double-blind, Placebo-controlled Phase) were not eligible and could not be rescreened to enter the trial. |
| **Other Exclusion Criteria** | |
| 21. | Prisoners or subjects who were compulsorily detained (involuntarily incarcerated) for treatment of either a psychiatric or physical (eg, infectious disease) illness were not enrolled into this trial. |
| 22. | Subjects who had been hospitalized, including hospitalization for psychosocial reasons, for more than 30 days total in the last 90 days prior to entry into the Conversion Phase for subjects entering the Conversion Phase, or prior to the Oral Stabilization Phase for subjects entering the Oral Stabilization Phase directly after screening. |

© 2022 Taipale H et al. *JAMA Psychiatry*.

| Table 5.3.2-1 | Exclusion Criteria |
|---|---|
| **Exclusion Criteria Assessed Prior to Entry into the Oral Stabilization Phase** | |
| 23. | Electroconvulsive therapy within 180 days prior to entry into the Oral Stabilization Phase. |
| **Exclusion Criteria Assessed Prior to Entry into the IM Depot Stabilization Phase** | |
| 24. | Subjects who had not achieved stability criteria on oral aripiprazole for 4 consecutive weeks (2 consecutive bi-weekly visits) by Week 12 of the Oral Stabilization Phase or subjects who at Week 10 still did not meet stability criteria. |
| **Exclusion Criteria Assessed Prior to Entry into the Double-blind, Placebo-controlled Phase** | |
| 25. | Subjects who had more than one excursion from stability criteria after achieving a response to single-blind aripiprazole IM depot treatment at a point in the trial where they would not have been able to maintain stability for a minimum of 12 weeks without exceeding 36 weeks total in the IM Depot Stabilization Phase. |
| 26. | Subjects who had not achieved stability criteria on aripiprazole IM depot for 12 consecutive weeks (6 consecutive bi-weekly visits) by Week 36 of the IM Depot Stabilization Phase or who had consecutive excursions at Weeks 26 and 28. |
| 27. | Subjects who met any of the criteria for exacerbation of psychotic symptoms/impending relapse (see Section 2.2 for definition) prior to randomization. Such subjects were withdrawn from the IM Depot Stabilization Phase for lack of efficacy and not randomly assigned treatment in the Double-blind, Placebo-controlled Phase. |

Source: protocol

**Kern 2006**

Inclusion criteria
All patients in the study were outpatients who met the DSM-IV diagnostic criteria for schizophrenia or schizoaffective disorder, were between ages of 18 and 65, able to speak and understand English, were on a stable dose of an oral typical antipsychotic, risperidone, or quetiapine for at least 1 month, and had not been hospitalized for psychiatric treatment for at least 2 months before randomization.

Exclusion criteria
Exclusion criteria included current suicidality, neurological disorder (e.g., epilepsy), acute or unstable medical condition, a clinically significant laboratory test value, gastrointestinal resection or stapling that may interfere with study medication absorption, and alcohol- or substance-dependence within the past 3 months. Patients were also excluded if they had received aripiprazole in a prior clinical study, had taken a selective serotonin reuptake inhibitor within 2 weeks before screening, or if they had taken an investigational drug within 4 weeks before randomization.

**Koshikawa 2016**

Inclusion criteria
Thirty outpatients who were at least 20 years old were included in this study. All patients had been diagnosed with schizophrenia or schizoaffective disorder based on DSM-IV-TR criteria. The inclusion criteria were: (1) being in a nonacute phase of the disease, (2) having a PANSS total score of 120 or less, and (3) having received RLAI for 2 months or longer.

Exclusion criteria
The exclusion criteria were: (1) comorbid serious physical disorders, (2) active suicidal ideation, (3) a history of attempted suicide, (4) a history of drug or alcohol abuse, (5) mental retardation, (6) pregnancy, (7) current treatment with oral RIS or oral PAL, or (8) current treatment with multiple oral antipsychotics. Because patients with too low an intelligence quotient (IQ) may have difficulty understanding the contents of the trial questionnaire, the Japanese Adult Reading Test (JART)was used to assess patients' IQ.

**Kramer 2007**

Inclusion criteria
Men and women, with ages from 18 to 65 years, were enrolled, provided they (1) had a diagnosis of schizophrenia DSM-IV for at least 1 year and (2) were experiencing an acute episode of schizophrenia PANSS total score, 70–120). Patients were required to be physically healthy, capable of being compliant with self-administration of medication or have consistent help available throughout the study, and able to complete self-administered questionnaires.

© 2022 Taipale H et al. *JAMA Psychiatry.*

Exclusion criteria

Patients were excluded if they had a DSM-IV Axis I diagnosis other than schizophrenia, if they had a DSM-IV Axis I diagnosis of substance dependence (except nicotine or caffeine) within 6 months before screening, or if they were considered to have a significant risk of suicidal or aggressive behavior. In addition, patients were excluded if they had medical conditions that could potentially alter the absorption, metabolism, or excretion of the study medication; relevant history of significant or unstable disease; known allergic reactions to barbiturates, carbamazepine, lamotrigine, phenytoin, paliperidone, or risperidone; a previous lack of response to risperidone; used a depot antipsychotic within 120 days; exposure to experimental treatment within 90 days before screening; electroconvulsive treatment within 3 months before screening; or had involuntary admission to a psychiatric hospital. Women were excluded if pregnant, nursing, or planning to become pregnant. Patients were encouraged to abstain from the use of alcohol or illicit substances for the entire study.

**MacFadden 2010**

Inclusion criteria

OP's diagnosed with schizophrenia, not adequately benifiting from current AP, at least 2 relapses in the past, clinically stable for at least 2 months

Exclusion criteria

Key exclusion criteria included a screening PANSS total score of 100 or more; current hospitalization, major medication changes, or worsening of psychiatric symptoms within two months before study entry; or current treatment with clozapine or carbamazepine. Other exclusion criteria included depot antipsychotic treatment or evidence of alcohol or drug dependence (DSM-IV Axis I criteria) within six months before entry.

**Marder 2003**

Inclusion criteria

The clinical diagnosis by a research psychiatrist was verified with the Structured Clinical Interview for DSM-IV Psychotic Disorders. All subjects were 18–60 years of age; had at least two documented episodes of acute schizophrenic illness or at least 2 years of continuing psychotic symptoms; had been outpatients for at least 1 month; and were considered candidates for maintenance therapy with an antipsychotic. Subjects were outpatients from the West Los Angeles Health Care Center of the Veterans Administration (VA) Greater Los Angeles Health Care System, the VA Long Beach Medical Center, or the UCLA Neuropsychiatric Hospital. + patients entered a 2-month stabilization phase

Exclusion criteria

Not specified

**Marder 2007**

Inclusion criteria

Stable outpatients - Eligible participants were 18-65 years old, living in the community, not currently competitively employed and interested in finding competitive work. Participants had a diagnosis of schizophrenia or schizoaffective disorder made by a research psychiatrist using DSM-IV criteria, and verified by the Structured Clinical Interview for DSM-IV (SCID). The IPS model of supported employment used in this study encourages inclusion of interested patients regardless of psychiatric symptom severity. Therefore, while all participants were clinically stable outpatients at the point of entry into the study, there was no exclusion for severity of psychotic symptoms.

Exclusion criteria

Exclusion criteria included organic brain disease, mental retardation, active substance dependence within the prior 6 months, or a chronic medical illness which would prevent employment.

**McEvoy 2014**

Inclusion criteria

Patients were adults aged 18 to 65 years with a diagnosis of schizophrenia or schizoaffective disorder as defined by criteria from the DSM-IV-TR and confirmed by the Structured Clinical Interview for DSM-IV. Patients were eligible if judged by their clinician and study psychiatrist as likely to benefit from treatment with paliperidone palmitate or haloperidol decanoate and to be at risk of efficacy failure based on a history of medication

© 2022 Taipale H et al. *JAMA Psychiatry.*

noncompliance, sig- nificant substance abuse, or both. All patients demonstrated adequate decisional capacity to participate and provided written informed consent.

Exclusion criteria

Patients with the following characteristics were excluded: currently stable and doing well using an antipsychotic regimen; not expected to benefit from the study medications due to past experience with risperidone, haloperidol, or paliperidone due to adverse effects or no improvement of severe symptoms in spite of an adequate treatment trial of at least 6 weeks' duration; moderate or severe tardive dyskinesia; presence of any medical condition that might preclude safe completion of the study; or intellectual disability. Women who were pregnant or breastfeeding were also excluded.

**Naber 2015**

Inclusion criteria

The study included stable, i.e., not acutely psychotic, patients needing a change from current oral antipsy- chotic treatment due to inadequate response, poor tolerability, or lack of adherence and who, in the judgment of the investigator, would benefit from LAI treatment. A CGI-S score ≥3 (mildly ill) and ≤5 (markedly ill) was required at both the screening and baseline visits, and treatment with oral antipsychotics was required for 3 months prior to screening.

Exclusion criteria

Patients with a diagnosis of psychiatric disorder or Axis I disorder (DSM-IV-TR criteria) other than schizophrenia, substance use disorder (except nicotine) that according to the investigator's judgment could compromise the patient's compliance with the study procedures, intolerance to or previous lack of efficacy with oral aripiprazole, risperidone, or paliperidone, or previous treatment with LAIs within 6 months prior to screening were excluded from participation in the study. Based on disease severity criteria, patients exhibiting acute exacerbation of psychotic symptoms, hospitalization for N3 months at the time of the screening visit, at significant risk of harming self or others, refractoriness to antipsychotic treatment, or with a history of failure to respond to/responding only to clozapine treatment were also excluded.

**NCT00191555**

Inclusion criteria

Schizophrenic patient meeting the DSM-IV diagnostic criteria more than one year ago and treated with antipsychotic for at least 1 year. Outpatient (or patient admitted to hospital for social or logistic reasons). Patient receiving a stable dose of the same conventional antipsychotic for at least 8 weeks before visit 1. Patient presenting a PANSS score equal or greater than 49 at Visit 2. Patient considered by the investigator as possible candidates for a switch, owing to inadequate efficacy or poor safety of the current treatment.

Exclusion criteria

Patient presenting a schizophreniform or a schizo-affective disorder according to the DSM-IV diagnostic criteria, or any other disorder from Axis I of the DSM-IV, or a disorder from Axis II (limit personality), substance dependence or substance abuse. Administration of an atypical antipsychotic drug during the 8 weeks preceding V1. History of resistance to antipsychotic drugs. Hospitalization in a psychiatric unit or in a psychiatric emergency department within the 8 weeks preceding the beginning of the study. Presence of serious unstable disease, such as a fatal outcome or hospitalization in an intensive care unit, is foreseeable within a period of 6 months.

**NCT00236379**

Inclusion criteria

Patients with a diagnosis of schizophrenia or schizoaffective disorder between the ages of 18 and 65 who were considered medically and psychiatrically (CGI < 4) stable and who would benefit from treatment with an atypical antipsychotic were included.

Exclusion criteria

Main exclusion criteria included patients that received risperidone, olanzapine, quetiapine, or clozapine within 30 days of baseline and any patient with a known history or diagnosis at screening/baseline visit of diabetes mellitus, or any illnesses that would bias study evaluations (including patients that were HIV positive at screening). Subjects with a positive history of diabetes or substance abuse (excluding nicotine and cannabis), significant medical illness, or unstable psychiatric disease were excluded. Subjects with a history of clozapine treatment for >4 consecutive weeks continuously within 3 months prior to the screening visit were excluded.

© 2022 Taipale H et al. *JAMA Psychiatry*.

**NCT00704509**

<u>Inclusion criteria</u>
Primary diagnosis of schizophrenia (DSM-IV-TR), between 18 and 65 years of age, experiencing clinically significant symptoms [CGI >= 4 (moderately ill), PANSS >= 60, score <=4 on PANSS items P2, P7,G8 at screening and baseline]; in the post-acute maintenance phase of the disease, not experiencing an acute exacerbation + antipsychotic medication unchanged within 8 weeks prior to screening or 4 weeks prior to baseline.

<u>Exclusion criteria</u>
Subjects were excluded if they were found to be at significant risk of suicide, treatment resistant, had experience an acute exacerbation within 8 weeks prior to screening, was unlikely to comply with the protocol or had a current diagnosis or a history of substance abuse.

**PEARL**

<u>Inclusion criteria</u>
Patients aged 18–75 years, inclusive, diagnosed with schizophrenia (based on DSM-IV-TR criteria), and experiencing an acute exacer- bation were eligible for enrollment. Entry criteria included a PANSS total score of ⩾80 with a score ⩾4 (moderate severity) on one or more positive subscale items, and a CGI-S scale score of ⩾4 (moderate severity).

<u>Exclusion criteria</u>
Patients were excluded from study participation if they were diagnosed with another Axis I or II disorder that had been a primary focus of treatment during the previous three months, if they had a history of treatment resistance to antipsychotic agents, or if they showed evidence of current or recent substance abuse or suicidal ideation.

**9.3.1.2. Exclusion Criteria**

Note: If any laboratory exclusion criteria were outside the normal range, the site could have the subject retested. If upon retesting the value remained outside the normal range, the significance of this value was to be discussed with the Medical Monitor for enrollment consideration.

1. Subject had a DSM-IV Axis I or Axis II diagnosis other than schizophrenia that had been the primary focus of treatment within 3 months of screening.
2. Subject answered "yes" to "Suicidal Ideation" item 4 (active suicidal ideation with some intent to act, without specific plan) or item 5 (active suicidal ideation with specific plan and intent) on the Columbia Suicide Severity Rating Scale (C-SSRS) assessment at screening (in the past month) or baseline.
3. Subject had attempted suicide within 3 months prior to the screening phase.
4. Subject had a current clinically significant medical condition including the following: neurological, metabolic (including Type 1 diabetes), hepatic, renal, hematological, pulmonary, cardiovascular, gastrointestinal, and/or urological disorder such as unstable angina, congestive heart failure (uncontrolled), or central nervous system (CNS) infection that would pose a risk to the subject if they were to participate in the study or that might confound the results of the study. Subjects with human immunodeficiency virus (HIV) seropositivity (or history of seropositivity) were excluded.

   **Note:** Active medical conditions that were minor or well-controlled were not exclusionary if they did not affect risk to the subject or the study results. In cases in which the impact of the condition upon risk to the subject or study results was unclear, the Medical Monitor was to be consulted. Any subject with a known

© 2022 Taipale H et al. *JAMA Psychiatry*.

cardiovascular disease or condition (even if controlled) was to be discussed with the Medical Monitor before being screened.

5. Subject had evidence of any chronic organic disease of the CNS such as tumors, inflammation, and active seizure disorder, vascular disorder, Parkinson's disease, Alzheimer's disease or other forms of dementia, myasthenia gravis, or other degenerative processes. In addition, subject was not to have a history of mental retardation or persistent neurological symptoms attributable to serious head injury.

   **Note:** Past history of febrile seizures, drug-induced seizures, or alcohol withdrawal seizures was not exclusionary.

6. Subject demonstrated evidence of acute hepatitis, clinically significant chronic hepatitis, or evidence of clinically significant impaired hepatic function through clinical and laboratory evaluation.

   **Note:** Subjects with serum alanine transaminase (ALT) or aspartate transaminase (AST) levels ≥ 3 times the upper limit of the reference ranges provided by the central laboratory **required** retesting. If on retesting, the laboratory value remained ≥ 3 times the upper limit, such subjects were discussed with the Medical Monitor for enrollment consideration.

7. Subject had a history of stomach or intestinal surgery or any other condition that could interfere with or was judged by the Investigator to interfere with absorption, distribution, metabolism, or excretion of study drug.

8. Subject with Type 1 or Type 2 insulin-dependent diabetes.

9. Subject with newly diagnosed Type 2 diabetes during screening. Subject with Type 2 diabetes was eligible for study inclusion if the following condition was met at screening:

   if a subject was currently being treated with oral anti-diabetic medication(s), the dose had to be stable for at least 4 weeks prior to screening. Such medication may be adjusted or discontinued during the study, as clinically indicated.

10. Subject had any abnormal laboratory parameter at screening that indicated a clinically significant medical condition as determined by the Investigator. Subjects with a fasting blood glucose at screening ≥ 126 mg/dL (7.0 mmol/L) or Hemoglobin A1c ($HbA_{1c}$) ≥ 7.0% were excluded.

    **Note:** Subjects with random (non-fasting) blood glucose at screening ≥ 200 mg/dL (11.1 mmol/L) were to be retested in a fasted state.

11. Subject had a prolactin concentration > 100 ng/mL at screening or had a history of pituitary adenoma.

12. Subject had a history of malignancy < 5 years prior to signing the informed consent, except for adequately treated basal cell or squamous cell skin cancer or *in situ* cervical cancer. Pituitary tumors of any duration were excluded.

13. Subject was judged to be resistant to antipsychotic treatment defined as any one of the following:

© 2022 Taipale H et al. *JAMA Psychiatry*.

f. failure to respond to > 2 marketed antipsychotic agents, given at an adequate dose and for an adequate duration (within the past 2 years)
g. history of treatment with clozapine for refractory psychosis

14. Subject was unlikely to achieve a stable condition for ≥ 12 weeks during the open-label lurasidone phase based on the totality of evidence from the psychiatric history and/or the current presentation.
15. Subject was receiving an antipsychotic medication above the maximum recommended (country-specific) dose at or prior to screening and, in the judgment of the Investigator, was unlikely to respond to standard doses of lurasidone.
16. Subject had received depot antipsychotics unless the last injection was at least one treatment cycle or at least 30 days (whichever is longer) prior to the screening phase.
17. Subject had received treatment with monoamine oxidase (MAO) inhibitors within 14 days prior to the screening phase.
18. Subject required treatment with any potent CYP3A4 inhibitors or inducers during the study (Appendix 3 of protocol [Appendix 16.1.1]).
19. Subject had received electroconvulsive therapy (ECT) treatment within the 3 months prior to screening or was expected to require ECT during the study.
20. Subject had a history of neuroleptic malignant syndrome.
21. Subject exhibited evidence of severe tardive dyskinesia, severe dystonia, or any other severe movement disorder. Severity was determined by the Investigator.
22. Subject had a history of alcohol or substance abuse (DSM-IV-TR criteria) within 3 months prior to screening or alcohol or substance dependence (DSM-IV-TR criteria) within 12 months prior to screening. The only exceptions included caffeine or nicotine abuse/dependence.
23. Subject tested positive for drugs of abuse at screening. In the event a subject tested positive for cannabinoids (tetrahydrocannabinol), the Investigator evaluated the subject's ability to abstain from using this drug during the study. This information was discussed with the Medical Monitor prior to study enrollment.
24. Subject had a history or presence of an abnormal electrocardiogram (ECG), which in the Investigator's opinion was clinically significant (Medical Monitor could be consulted to determine clinical significance).
25. Subject had poor peripheral venous access that would limit the ability to draw blood as judged by the Investigator.
26. Subject had a history of hypersensitivity to more than 2 distinct chemical classes of drug (eg, sulfas and penicillins).
27. Subject was screened or washed out previously more than 3 times for this study.
28. Subject was currently participating, or had participated in, a study with an investigational or marketed compound or device within three months prior to signing the informed consent, or had participated in two or more studies within 12 months prior to signing the informed consent.
29. Subject was homeless or did not have a stable residence for the three months prior to the screening phase.
30. Subject was unable to cooperate with any study procedures, unlikely to adhere to the study procedures and keep appointments, in the opinion of the Investigator, or was planning to relocate during the study.
31. Subject required guardianship under the laws of his/her country.

© 2022 Taipale H et al. *JAMA Psychiatry.*

**Peuskens 2007**

Inclusion criteria

Patients from centers in Bulgaria, India, Poland, Russia, and Ukraine were considered eligible for inclusion in the study if they were ages ≥18 to ≤65 years; had a documented clinical diagnosis of schizophrenia (according to DSM-IV) for at least two years; were clinically stable before entering the stabilization phase (defined as a CGI-S score ≤4 and unchanged treatment [both compound and dose] with antipsychotic agent[s] within four weeks prior to entering the study); and had a PANSS total score ≤60 at enrollment (Week 16).

Exclusion criteria

Exclusion criteria included treatment with depot antipsychotics within one dosing interval before enrollment (Week 16); pregnancy or breastfeeding; any DSM-IV Axis 1disorder not defined in the inclusion criteria; any clinically significant deviations from the reference range in clinical laboratory test results at enrollment, as evaluated by the investigator; intolerance or lack of response to quetiapine; previous treatment with clozapine and/or valproic acid within two months of enrollment; and history of nonadherence, as judged by the investigator.

**Pigott 2003**

Inclusion criteria

Men and women aged ≥ 18 years were enrolled in the study. All were required to have a confirmed diagnosis of schizophrenia, defined by DSM-IV criteria. Diagnoses must have been made at least 2 years prior to entry, and continued antipsychotic treatment during this period was required to classify diagnoses as chronic. Each patient's condition at entry had to be stable (no significant improvement or worsening of symptoms within the past 3 months). The term "stable" refers to a consistency of residual symptomatology over the past 3 months and does not include those patients doing well or controlled on treatment with current medication. Patients enrolled in the study were still experiencing significant symptomatology as evidenced by a mean baseline PANSS total score of 81.8 and a CGI-S score of 3.5. All patients had to be receiving antipsychotic treatment at entry and must have shown a response to this treatment. Patients were required to have a PANSS score of at least 60 and a score of not more than 4 (moderate) on the subscale for hostility or uncooperativeness. Patients also had to score no more than 4 (moderately ill) on the CGI-S. Women of childbearing potential were enrolled only if they had tested negative for pregnancy and were using a reliable form of contraception.

Exclusion criteria

Patients were excluded from the study if they were experiencing acute relapse; had a psychiatric disorder other than schizophrenia; had a history of or presented with de- lirium, dementia, amnesia, or a cognitive disorder; were known to be treatment resistant to antipsychotics; had re- ceived fluoxetine within 4 weeks of randomization; were dependent on benzodiazepines or had a history of alcohol or substance abuse; or were receiving a long-acting antipsychotic and the last dose had been administered less than 1 full cycle plus 1 week prior to randomization. Patients were also excluded if they presented with a significant risk of suicide, a history of neuroleptic malignant syndrome, thyroid pathology, or hypersensitivity to aripiprazole or other quinolinones; if they had enrolled in an aripiprazole clinical study or any clinical trial with an investigational agent within the last month; or if they had been exposed to electroconvulsive therapy within 2 months of randomization.

© 2022 Taipale H et al. *JAMA Psychiatry*.

### 5.2.2 Exclusion Criteria

Patients who met any of the following criteria were excluded from the study:

1) Currently in an acute relapse;
2) Axis I (DSM-IV) diagnosis of schizoaffective or bipolar disorder;
3) Presented with a clinical picture and/or history that was consistent with delirium, dementia, amnestic or other cognitive disorder;
4) Considered treatment-resistant to neuroleptic medication (patients needed to have shown a previous response to a neuroleptic medication other than clozapine);
5) Treated with a long-acting antipsychotic in which the last dose was less than one full cycle plus 1 week prior to randomization (ie, haloperidol decanoate treatment within the past 5 weeks or fluphenazine decanoate treatment within the past 3 weeks);
6) Treated with fluoxetine within the 4 weeks prior to randomization;
7) Previously enrolled in an aripiprazole clinical study;
8) Dependent on benzodiazepines (low doses, eg, 1 to 2 mg/day of lorazepam or 2.5 to 10 mg of diazepam used only nightly for sleep was acceptable);
9) Met DSM-IV criteria for any significant substance use disorder within the past 3 months;
10) Detectable levels of cocaine in the drug screen or a positive blood alcohol level (could be reassessed prior to randomization, if necessary). Patients with a positive drug screen for stimulants or other drugs of abuse were to be discussed with the medical monitor of the CRO prior to randomization;
11) Represented a significant risk of committing suicide based on history or mental status examination;
12) History of neuroleptic malignant syndrome;
13) Likely to have required prohibited concomitant therapy during the trial;
14) Known to be allergic or hypersensitive to aripiprazole or other quinolones;
15) Unstable thyroid pathology within the past 3 months prior to randomization;
16) History or evidence of a medical condition that would have exposed the patient to an undue risk of a significant adverse event (AE) or would have interfered with assessments of safety or efficacy during the course of the trial;
17) Clinically significant abnormal laboratory test results (including urine drug screen), vital sign, or ECG findings;
18) Pregnant or nursing women, or women of childbearing potential not using adequate contraception or who were judged to be unreliable in their use of contraception;
19) Participation in any clinical trial with an investigational agent within one month prior to ransomization;
20) Electroconvulsive treatment within 2 months prior to randomization unless permission was obtained from BMS.

**Purdon 2000**

Inclusion criteria

The diagnosis of schizophrenia as defined by DSM-IV was confirmed on clinical interview by the principal investigator at each site. The sample included men and women aged 18 to 65 years who were within 5 years of their first exposure to neuroleptic treatment and had symptom severity at least in the mild range.

© 2022 Taipale H et al. *JAMA Psychiatry.*

Exclusion criteria
participants were excluded from the study if they were pregnant or lactating, had prior medical histories of central nervous system disease or severe head injury, or if they had active serious illness or substance abuse disorders in the previous 30 days.

**Rémillard 2005y1**

Inclusion criteria
Thirty-one outpatients with a diagnosis of schizophrenia (DSM-III criteria) participated in the study.

Exclusion criteria
Exclusion criteria for all participants included a history of drug or alcohol abuse or neurological dis- ease. Written informed consent was obtained from all participants.

**REPRIEVE**

Inclusion criteria
Patients must agree to cooperate with all tests and examinations required by the protocol, be willing to comply fully with treatment and able to ingest oral medication. Patients must understand the nature of the study and must sign an informed consent document. Patients will have a clear diagnosis of schizophrenia according to DSM-IV criteria for at least 1 year. Patients must need of ongoing psychiatric treatment and must have a documented reason why a change in treatment is needed which might lead to a clinical improvement. At screening, patients will have a PANSS of no more than 100 and a CGI of no more than 5 (i.e. must not be severely ill or worse). Patients must be outpatients at the time of screening and have not been an inpatient to treat schizophrenia for at least 1 week prior to the screening visit. Patients must have a history of at least 2 prior episodes of relapse or impending relapse in the 2 years preceding the screening visit.

Exclusion criteria
Pregnant or nursing (lactating) women, or women who plan on conceiving during the course of the study. Patients who meet the DSM-IV criteria for schizophreniform disorder (295.40) and schizoaffective (295.70). Patients with active symptoms of any other primary psychiatric diagnosis (Axis I) or prominent Axis II disorder which would interfere with compliance to the protocol. Patients who have a diagnosis or history suggestive of chemical dependence, or drug-induced toxic psychosis in the preceding 6 months; diagnosis or history of abuse (except for nicotine and caffeine) within the past 3 months, or a clinical presentation possibly confounded by the use of recreational drugs or alcohol. Patients who have a positive urine drug screen (at the screening visit). If opiates are positive at screening and clearly due to the use of pain killing medication, the patient may be re screened after the medication has been discontinued and enrolled in the study if urine drug screen is negative. Note: Occasional users of recreational drugs other than cocaine, amphetamines, hallucinogens, or parenteral drugs may be recruited. Patients who are dependent on nicotine, caffeine, or theophylline are allowed to enter the study. Patients who are mentally disabled (moderate to severe). Patients who have had a history of being in a coma for more than 24 hrs. Patients who have had thoughts of committing suicide within 6 months prior to screening or at baseline or suicide behaviors within 2 years prior to screening or at baseline. Patients thought to be of imminent risk of harm to others or in imminent legal difficulty. Patients under any form of legal compulsion to remain hospitalized or undergo treatment or assessment. Patients who have any disability that prevent them from completing any of the study requirements. Patients with a known clinically significant ECG abnormality including PR interval >240 msec, QRS complex >110 msec, QTcF >=450 msec, or congenital long QT syndrome based on central ECG reading results. Treatment naive, first episode patients. Patients taking iloperidone at the screening visit or with a known hypersensitivity to drugs chemically related to benzioxazoles. Note: Active medical conditions that are minor or well-controlled are not exclusionary if they do not affect risk to the patient or the study results.

**RIS JPN S31**

Inclusion criteria
Patients who met all of the following inclusion criteria and none of the exclusion criteria were enrolled in this study. (1) Patients diagnosed with schizophrenia according to the criteria of Diagnostic and Statistical Manual of Mental Disorders, 4th Edition (DSM-IV) (295.30, 295.10, 295.20, 295.90, 295.60). (2) Patients who are taking antipsychotics at a risperidone-equivalent dose up to 6 mg/day for 28 days before the date of informed consent with no change in the dosage and administration (except for medications used on an as-needed basis). (3) Patients with a total PANSS score of $\geq 60$ to $< 120$. (4) Patients who are at least 20 years of age on the day of informed consent. (5) Both inpatients and outpatients are acceptable (patient's discharge during the study period is allowed.) (6) Patients who can give their own consent in writing to participate in the study. (If it is

© 2022 Taipale H et al. *JAMA Psychiatry*.

objectively considered difficult to obtain the patient's own consent, it is allowed to obtain consent of the patient's representative.)

Exclusion criteria
Diagnosis of mental disease other than schizophrenia treated with a sustained-release injection of other antipsychotic medications within 60 days before the initiation of the study history of cerebrovascular accident, convulsive disorder such as epilepsy, diabetes mellitus, liver disease, kidney disease, cardiovascular disorder, malignancy or physical exhaustion due to dehydration or malnutrition have risk factors of diabetes mellitus, such as hyperglycemia.

**Rui 2014**

Inclusion criteria
Patients of either sex, aged ≥18 years, diagnosed with schizophrenia based on DSM-IV-TR for at least 1 year before screening, and a PANSS total score between 70 and 120 (inclusive), at screening and baseline were eligible for enrollment.

Exclusion criteria
Major exclusion criteria for the study included: drug dependence (excluding nicotine and caffeine dependence) within 6 months before screening according to DSM-IV, history of cardiovascular, respiratory, neurologic, renal, hepatic, endocrine, or immunologic diseases, presence of circumstances that may increase the risk of the occurrence of torsade de pointes or sudden death, heart rate b 50 bpm, presence of congenital prolongation of the QT interval or demonstration of repeated prolonged QTc Fridericia interval N 450 ms in N1 electrocardiogram (ECG), neuroleptic malignant syndrome and hypersensitivity to risperidone, paliperidone, or their excipients. Patients treated with clozapine refractory or treatment resistant schizophrenia, monoamine oxidase inhibitor antidepressants within 4 weeks before screening, depot antipsychotic drugs within 120 days, paliperidone palmitate within 10 months or electroconvulsive therapy within 60 days before screening, and pregnant and lactating women were all excluded from the study.

### 3.2.3. Exclusion Criteria

Potential subjects who met any of the following criteria were excluded from participating in this study:

- A drug dependence diagnosis according to DSM-IV-TR (excluding nicotine and caffeine dependence) within 6 months before screening.
- Any medical condition that could change the absorption, metabolism, or elimination of drug, including Crohn's disease and hepatic or renal diseases.
- Relevant history of any significant and/or unstable cardiovascular, respiratory, neurologic (including seizures or significant cerebrovascular dysfunction), renal, hepatic, endocrine, or immunologic diseases.

© 2022 Taipale H et al. *JAMA Psychiatry.*

- History or presence of circumstances that could increase the risk of the occurrence of torsade de pointes or sudden death in association with the use of drugs that prolong the corrected QT (QTc) interval, including:
  - Heart rate (HR) <50 beats per minute (bpm).
  - Demonstration of repeated prolonged QTc Fridericia interval >450 ms, as measured on more than one ECG (either during screening, or from prior medical record).
  - The following cardiac conditions: sick sinus syndrome, complete atrioventricular block, congestive heart failure, and polymorphic ventricular tachycardia.
  - Clinically relevant hypocalcemia, hypokalemia, or hypomagnesemia.
  - Concomitant use of drugs that prolong the QTc interval (including Class Ia (eg, quinidine, procainamide) or Class III (eg, amiodarone, sotalol) antiarrhythmic medications).
  - Presence of congenital prolongation of the QT interval (Romano-Ward Syndrome, Jervell and Lange-Nielsen syndrome.
- Past history of neuroleptic malignant syndrome.
- Known or suspected Stevens Johnson Syndrome after exposure to phenytoin, carbamazepine, barbiturates, or lamotrigine.
- An allergy or hypersensitivity to risperidone, paliperidone, or their excipients;
- History of severe gastrointestinal narrowing (pathologic or iatrogenic).
- History of no response to risperidone or paliperidone when psychotic or acutely psychotic. Lack of response was defined as subjects who have had (at least twice) a documented medical history of no clinical response, despite adequate doses and durations of treatment, or the inability to tolerate effective doses.
- Exposure to clozapine for treatment refractory or treatment resistant schizophrenia.
- Participation in other studies within 90 days before screening.
- Significant risk of suicide or homicidal behavior, or significant risk of deliberate self-harm or harm to others. This was based upon an investigator's judgment after his/her review of the C-SSRS, subject history and clinical relevance.
- Pregnant or breast-feeding females.
- Alanine aminotransferase (ALT) or aspartate aminotransferase (AST) levels >2 times the upper limit of normal (ULN) at screening.
- White blood cell (WBC) count below the lower limit of the central laboratory reference range at the screening visit.
- Injection of depot antipsychotic drugs within 120 days or took paliperidone palmitate within 10 months before screening.
- Exposure to MAOI antidepressants within 4 weeks before screening.
- Treatment with electroconvulsive therapy (ECT) within 60 days before screening.
- Employees of the investigator or study site, with direct involvement in the proposed study or other studies under the direction of that investigator or study site, or who were family members of the employees or the investigator.
- For purposes of emergency psychiatric evaluation, subjects who were unable to readily access the investigational site via telephone or in person within a reasonable time period were not permitted to participate in the study.

© 2022 Taipale H et al. *JAMA Psychiatry*.

**Shrivastava 2000**

Inclusion criteria
Patients suffering from schizophrenia and admitted to the hospital for acute exacerbation were selected for the study. During the acute stage, all were treated with haloperidol over a period of 2 to 4 weeks

Exclusion criteria
Not specified

**Subotnik 2015**

Inclusion criteria
(1) A recent onset of psychotic illness, with the beginning of the first major psychotic episode within the past 2 years, and (2) a diagnosis using the DSM-IV of schizophrenia, schizoaffective disorder, depressed type, or schizophreniform disorder. Additional criteria: 1) 18 to 45 years of age; 2) no evidence of a known neurological disorder; 3) no evidence of significant and habitual drug abuse or alcoholism in the 6 months prior to hospitalization and no evidence that the psychosis was accounted for by substance abuse; 4) no premorbid mental retardation; 5) sufficient acculturation and fluency in the English language to avoid invalidating research measures; 6) residence within commuting distance of the UCLA Aftercare Research Program; and 7) treatment with risperidone was not contraindicated.

Exclusion criteria
We excluded individuals with substantial substance abuse.

**Vontour 2005**

Inclusion criteria
DSM-IV diagnosis of schizophrenia or schizoaffective disorder, between ages of 18 and 52 years, and on olanzapine for a minimum of 3 months prior to participation. They had to had an outpatient status for at least 3 months, as well as vision and hearing intact or corrected to extent that will allow participation in vocational training and cognitive testing. Participants also had to score in impaired range on at least one test from a cognitive battery designed to be sensitive to impairments in schizophrenia. Participants had to have the ability to participate in the informed consent process, as evidenced by an assessment of the capacity to give consent for research developed by the Maryland Psychiatric Research Center.

Exclusion criteria
History of head injury, mental retardation or neurological disorder. Having below a 4th grade reading level (32) according to the WRAT-3. Taking multiple atypical antipsychotics. Taking any decanoate antipsychotic Hospitalization in last 3 months. Employment. Alcohol or drug abuse that interferes with functioning or medication compliance.

**Wang 2006**

Inclusion criteria
To be eligible, patients had to be judged by their treating clinician to have been stable on conventional antipsychotic medication for at least 2 years. All subjects (in consultation with their treating staff) were required to have a reason for switching to atypical antipsychotic medication including a desire for improved efficacy, improved side effect profile and/or reduced risk of developing or worsening tardive dyskinesia.

Exclusion criteria
Patients with a previous therapeutic trial with an atypical antipsychotic medication were excluded. Patients with unstable psychiatric, metabolic, hematologic, cardiovascular, hepatic or renal function were excluded from the study.

© 2022 Taipale H et al. *JAMA Psychiatry*.

## eReferences


1. Schneider-Thoma J, Chalkou K, Dörries C, Cipriani A, Leucht S. Comparative efficacy and tolerability of 31 oral and long-acting injectable antipsychotics for maintenance treatment of adults with schizophrenia: a systematic review and network meta-analysis. *Lancet*. 2021.
2. World Health Organization. International Classification of Diseases (ICD-10). https://icd.who.int/browse10/2010/en. Published 2010. Accessed April 21, 2020.
3. Tiihonen J, Mittendorfer-Rutz E, Majak M, et al. Real-world effectiveness of antipsychotic treatments in a nationwide cohort of 29 823 patients with schizophrenia. *JAMA Psychiatry*. 2017;74(7):686-693. doi:10.1001/jamapsychiatry.2017.1322
4. Taipale H, Mittendorfer-Rutz E, Alexanderson K, et al. Antipsychotics and mortality in a nationwide cohort of 29,823 patients with schizophrenia. *Schizophr Res*. 2018;197:274-280. doi:10.1016/j.schres.2017.12.010
5. Taipale H, Tiihonen J. Registry-based studies: What they can tell us, and what they cannot. *Eur Neuropsychopharmacol*. 2021;45:35-37. doi:10.1016/j.euroneuro.2021.03.005
6. Ceraso A, Lin JJ, Schneider-Thoma J, et al. Maintenance treatment with antipsychotic drugs for schizophrenia. *Cochrane Database Syst Rev*. 2020;2020(8). doi:10.1002/14651858.CD008016.pub3
7. Tiihonen J, Tanskanen A, Taipale H. 20-year nationwide follow-up study on discontinuation of antipsychotic treatment in first-episode schizophrenia. *Am J Psychiatry*. 2018;175(8):765-773. doi:10.1176/appi.ajp.2018.17091001


© 2022 Taipale H et al. *JAMA Psychiatry*.

## eResults.

### Primary outcome in subpopulations ineligible for specific reasons (continued from Results section in main manuscript)

Ineligible individuals >65 years in the Finnish cohort but not in the Swedish cohort had decreased risk of hospitalisation due to psychosis compared to the eligible population, i.e., individuals being 18-65 years old (HR 0.71 (0.61-0.83); Table 4). In both cohorts, higher risks were found for those with a history of substance abuse (HR 1.43 (1.29-1.59) and 1.88 (1.61-2.21)), suicide attempt (1.61 (1.42-1.83) and 2.13 (1.79-2.54)), treatment resistance (1.71 (1.52-1.93) and 2.31 (1.87-2.85)), or tardive dyskinesia (1.77 (0.95-3.31) and 2.13 (1.36-3.32)) compared to eligible individuals. These differences were more pronounced in the Swedish cohort.

After stratifying ineligible individuals according to specific exclusion criteria, we found that the individuals with the highest rates of hospitalisation due to psychosis within 12 months were those with treatment-resistance (risk 26% in the Finnish and 30% in the Swedish cohort), tardive dyskinesia (25% and 28%), a history of suicide attempts (23% and 26%), or substance abuse (21% and 24%; Table 4). When stratified by the number of exclusion criteria fulfilled, the risks of hospitalisation due to psychosis increased with increasing numbers of exclusion criteria in both countries, compared to eligible individuals (eTable 2). Individuals who were excluded due to MS/AD use mainly had a diagnosis of schizophrenia although schizoaffective disorder was relatively more common in this ineligibility subgroup (eTable 3).

© 2022 Taipale H et al. *JAMA Psychiatry*.

## Online-Only Tables

**eTable 1A. Basic characteristics of the Finnish cohort and overlap between specific exclusion criterion with each other. Proportions calculated for columns (e.g. of persons excluded due to criterion “Age,” 10.4% (N=186) had diagnosis of substance abuse).**

| | Age % (N) | Substance abuse % (N) | Suicide attempt % (N) | Treatment resistance % (N) | Serious somatic disease, broader, % (N) | Serious somatic disease, narrower, % (N) | MS/AD initiation % (N) | Intellectual disability % (N) | Tardive dyskinesia % (N) | Pregnant/ breastfeeding women % (N) |
|---|---|---|---|---|---|---|---|---|---|---|
| **Basic characteristics** | | | | | | | | | | |
| % of entire cohort (N) | 10.1 (1795) | 21.4 (3808) | 9.5 (1690) | 10.1 (1805) | 40.5 (7202) | 28.6 (5087) | 44.9 (7983) | 3.5 (622) | 0.2 (40) | 0.8 (143) |
| Male sex | 38.3 (687) | 65.9 (2510) | 45.2 (763) | 57.1 (1030) | 44.9 (3231) | 53.4 (2718) | 45.8 (3657) | 49.2 (306) | 55.0 (22) | NA |
| Mean age (SD) | 67.8 (6.7) | 42.6 (13.1) | 41.9 (13.1) | 42.0 (13.1) | 50.7 (13.8) | 50.1 (14.1) | 47.4 (13.5) | 49.8 (12.9) | 46.4 (14.9) | 32.3 (6.4) |
| **Overlap between groups** | | | | | | | | | | |
| Age | | 4.9 (186) | 5.0 (84) | 5.1 (92) | 15.2 (1095) | 16.0 (812) | 8.9 (710) | 12.9 (80) | NA | NA |
| Substance use | 10.4 (186) | | 52.2 (882) | 30.5 (551) | 23.2 (1667) | 30.6 (1554) | 23.7 (1888) | 16.6 (103) | 35.0 (14) | 30.1 (43) |
| Suicide attempt | 4.7 (84) | 23.2 (882) | | 16.6 (299) | 11.3 (814) | 13.9 (708) | 12.6 (1009) | 6.1 (38) | 20.0 (8) | 20.0 (28) |
| Treatment resistance | 5.1 (92) | 14.5 (551) | 17.7 (299) | | 11.3 (810) | 12.8 (651) | 10.5 (834) | 8.7 (54) | 27.5 (11) | 7.0 (10) |
| Serious somatic disease, broader | 61.0 (1095) | 43.8 (1667) | 48.2 (814) | 44.9 (810) | | 66.5 (3382) | 46.0 (3669) | 48.2 (300) | 60.0 (24) | 35.0 (50) |
| Serious somatic disease, narrower | 45.2 (812) | 40.8 (1554) | 41.9 (708) | 36.1 (651) | 47.0 (3382) | | 32.6 (2602) | 36.3 (226) | 90.0 (36) | 25.9 (37) |
| MS/AD initiation | 39.6 (710) | 49.6 (1888) | 59.7 (1009) | 46.2 (834) | 50.9 (3669) | 51.2 (2602) | | 53.9 (335) | 60.0 (24) | 40.6 (58) |
| Intellectual disability | 4.5 (80) | 2.7 (103) | 2.3 (38) | 3.0 (54) | 4.2 (300) | 4.4 (226) | 4.2 (335) | | NA | NA |
| Tardive dyskinesia | NA | 0.4 (14) | 0.5 (8) | 0.6 (11) | 0.3 (24) | 0.7 (36) | 0.3 (24) | NA | | NA |
| Pregnant or breastfeeding women | NA | 1.1 (43) | 1.7 (28) | 0.6 (10) | 0.7 (50) | 0.7 (37) | 0.7 (58) | NA | NA | |

MS: mood stabiliser, AD: antidepressant, LAI: long-acting injectable antipsychotic. NA: cell count <5.

© 2022 Taipale H et al. *JAMA Psychiatry*.

**eTable 1B. Basic characteristics of the Swedish cohort and overlap between specific exclusion criteria with each other. Proportions calculated for columns (e.g. of persons excluded due to criterion "Age," 14.0% (N=55) had diagnosis of substance abuse).**

| | Age % (N) | Substance abuse % (N) | Suicide attempt % (N) | Treatment resistance % (N) | Serious somatic disease, broader, % (N) | Serious somatic disease, narrower, % (N) | MS/AD initiation % (N) | Intellectual disability % (N) | Tardive dyskinesia % (N) | Pregnant/ breastfeeding women % (N) |
|---|---|---|---|---|---|---|---|---|---|---|
| **Basic characteristics** | | | | | | | | | | |
| % of entire cohort (N) | 5.3 (394) | 24.5 (1828) | 13.8 (1032) | 6.0 (450) | 38.4 (2866) | 23.4 (1747) | 44.0 (3281) | 3.8 (282) | 1.0 (75) | 0.4 (30) |
| Male sex | 36.3 (143) | 67.3 (1230) | 48.1 (496) | 56.7 (255) | 47.5 (1362) | 59.4 (1038) | 49.9 (1638) | 55.7 (157) | 56.0 (42) | NA |
| Mean age (SD) | 68.1 (2.0) | 44.1 (12.2) | 43.7 (12.3) | 44.4 (12.9) | 48.4 (12.8) | 47.7 (13.1) | 46.0 (12.5) | 43.7 (13.3) | 47.0 (13.6) | 34.0 (4.8) |
| **Overlap between groups** | | | | | | | | | | |
| Age | | 3.0 (55) | 3.3 (34) | 4.2 (19) | 8.3 (238) | 8.3 (145) | 5.3 (175) | 5.3 (15) | 9.3 (7) | NA |
| Substance use | 14.0 (55) | | 51.7 (533) | 28.2 (127) | 28.3 (810) | 36.6 (640) | 26.4 (866) | 27.0 (76) | 25.3 (19) | 23.3 (7) |
| Suicide attempt | 8.6 (34) | 29.2 (533) | | 19.8 (89) | 17.3 (497) | 21.4 (374) | 18.4 (603) | 17.0 (48) | 17.3 (13) | 16.7 (5) |
| Treatment resistance | 4.8 (19) | 7.0 (127) | 8.6 (89) | | 7.3 (208) | 8.5 (148) | 6.3 (206) | 8.9 (25) | 12.0 (9) | NA |
| Serious somatic disease, broader | 60.4 (238) | 44.3 (810) | 48.2 (497) | 46.2 (208) | | 60.9 (1063) | 43.9 (1441) | 47.2 (133) | 62.7 (47) | 40.0 (12) |
| Serious somatic disease, narrower | 36.8 (145) | 35.0 (640) | 36.2 (374) | 32.9 (148) | 37.1 (1063) | | 26.8 (880) | 27.0 (76) | 90.7 (68) | 23.3 (7) |
| MS/AD initiation | 44.4 (175) | 47.4 (866) | 58.4 (603) | 45.8 (206) | 50.3 (1441) | 50.4 (880) | | 48.6 (137) | 48.0 (36) | 33.3 (10) |
| Intellectual disability | 3.8 (15) | 4.2 (76) | 4.7 (48) | 5.6 (25) | 4.6 (133) | 4.4 (76) | 4.2 (137) | | NA | NA |
| Tardive dyskinesia | 1.9 (7) | 1.0 (19) | 1.3 (13) | 2.0 (9) | 1.6 (47) | 3.9 (68) | 1.1 (36) | NA | | NA |
| Pregnant or breastfeeding women | NA | 0.4 (7) | 0.5 (5) | NA | 0.4 (12) | 0.4 (7) | 0.3 (10) | NA | NA | |

MS: mood stabiliser, AD: antidepressant, LAI: long-acting injectable antipsychotic, NA: cell count <5.

© 2022 Taipale H et al. *JAMA Psychiatry*.

**eTable 2. Risk of rehospitalisation within 12 months when stratified by the number of exclusion criteria met in comparison with eligible individuals.** Hazard Ratios (HR) with 95% CIs; a HR>1 means higher risk in the ineligible group.

| | Finnish cohort | | | | Swedish cohort | | | |
|---|---|---|---|---|---|---|---|---|
| **Criteria met** | **N (%)** | **% (N) relapsed** | **Mean time (days) to event/ censoring (SD)** | **HR 95% CI** | **N (%)** | **% (N) relapsed** | **Mean time (days) to event/ censoring (SD)** | **HR 95% CI** |
| 0 | 3580 (20.1) | 17.2 (615) | 278 (130) | reference | 1619 (21.7) | 14.8 (240) | 273 (127) | reference |
| 1 | 5875 (33.0) | 17.9 (1053) | 268 (134) | 1.07 (0.97-1.19) | 2514 (33.7) | 17.6 (442) | 261 (133) | 1.24 (1.06-1.45) |
| 2 | 5075 (28.5) | 17.7 (899) | 257 (137) | 1.10 (0.99-1.22) | 1987 (26.6) | 19.5 (388) | 249 (137) | 1.43 (1.22-1.68) |
| ≥3 | 3271 (18.4) | 20.1 (657) | 237 (141) | 1.32 (1.18-1.48) | 1338 (17.9) | 25.7 (344) | 224 (140) | 2.05 (1.74-2.42) |

© 2022 Taipale H et al. *JAMA Psychiatry*.

**eTable 3. Frequencies of mood stabiliser (MS) or antidepressant (AD) use among persons with schizophrenia or schizoaffective disorder.** MS/AD use was an exclusion criterion and proportions are calculated from the entire cohort.

| | **Finnish cohort** | | **Swedish cohort** | |
|---|---|---|---|---|
| | **Schizophrenia % (N)** | **Schizoaffective disorder % (N)** | **Schizophrenia % (N)** | **Schizoaffective disorder % (N)** |
| MS/AD users | 38.4 (5363) | 68.2 (2620) | 33.0 (1710) | 69.0 (1571) |

© 2022 Taipale H et al. *JAMA Psychiatry*.

**eTable 4. Additional analyses on eligibility of clozapine users.** The same RCT-derived inclusion and exclusion criteria were applied to clozapine users (with an exception of treatment resistance criteria) who otherwise were not included in the main analyses.

| | **Finnish dataset (N=7113)** | | **Swedish dataset (N=1678)** | |
|---|---|---|---|---|
| | **Eligible % (N)** | **Ineligible % (N)** | **Eligible % (N)** | **Ineligible % (N)** |
| Clozapine users | 18.4 (1307) | 81.6 (5806) | 19.8 (332) | 80.2 (1346) |
| **Most common specific reasons for ineligibility** | | | | |
| MS/AD use | NA | 61.9 (3596) | NA | 61.6 (829) |
| Somatic broad | NA | 55.7 (3234) | NA | 48.1 (648) |
| MS: mood stabiliser, AP: antipsychotic. | | | | |

© 2022 Taipale H et al. *JAMA Psychiatry*.

**eTable 5. Additional analyses on individuals only treated in outpatient care (N=4727): risk of hospitalisation due to psychosis in individuals ineligible (after application of ≥ 1 exclusion criteria, "any reason" and due to specific reasons) vs eligible (persons without any exclusion criterion) in the Swedish cohort and corresponding hazard ratios (HR) with 95% CIs**. A HR>1 means higher risk in the ineligible group.

| **N (%) of eligible/ ineligible** | **% (N) relapsed** | **Mean time (days) to event/ censoring (SD)** | **HR 95% CI, p-value** |
|---|---|---|---|
| **Any reason** | | | |
| Eligible N=1219 (25.8%) | 5.7 (70) | 278 (128) | 1.23 (0.95-1.61), p=0.1219 |
| Ineligible N=3508 (74.2%) | 6.7 (236) | 261 (134) | |
| **Age <18 and >65 years** | | | |
| Eligible N=1219 (25.8%) | 5.7 (70) | 278 (128) | 0.98 (0.52-1.85), p=0.9471 |
| Ineligible N=198 (4.2%) | 5.6 (11) | 274 (131) | |
| **Substance abuse** | | | |
| Eligible N=1219 (25.8%) | 5.7 (70) | 278 (128) | 1.55 (1.10-2.17), p=0.0117 |
| Ineligible N=829 (17.5%) | 7.7 (64) | 234 (139) | |
| **Suicide attempt** | | | |
| Eligible N=1219 (25.8%) | 5.7 (70) | 278 (128) | 2.13 (1.43-3.17), p=0.0002 |
| Ineligible N=364 (7.7%) | 10.2 (37) | 223 (142) | |
| **Treatment resistance** | | | |
| Eligible N=1219 (25.8%) | 5.7 (70) | 278 (128) | 1.91 (1.01-3.61), p=0.0462 |
| Ineligible N=105 (2.2%) | 10.5 (11) | 261 (137) | |
| **Serious somatic disease, broader definition** | | | |
| Eligible N=1219 (25.8%) | 5.7 (70) | 278 (128) | 1.27 (0.94-1.71), p=0.1198 |
| Ineligible N=1678 (35.5%) | 6.8 (114) | 256 (135) | |
| **Serious somatic disease, narrower definition** | | | |
| Eligible N=1219 (25.8%) | 5.7 (70) | 278 (128) | 1.24 (0.89-1.74), p=0.2028 |
| Ineligible N=1016 (21.5%) | 6.6 (67) | 250 (136) | |
| **Mood stabiliser/ antidepressant concomitant use** | | | |
| Eligible N=1219 (25.8%) | 5.7 (70) | 278 (128) | 1.11 (0.83-1.48), p=0.4954 |
| Ineligible N=2088 (44.2%) | 6.2 (128) | 266 (132) | |
| **Intellectual disability** | | | |
| Eligible N=1219 (25.8%) | 5.7 (70) | 278 (128) | 0.98 (0.49-1.96), p=0.9529 |
| Ineligible N=163 (3.4%) | 5.5 (9) | 269 (131) | |
| **Tardive dyskinesia** | | | |
| Eligible N=1219 (25.8%) | 5.7 (70) | 278 (128) | NA |
| Ineligible N=29 (0.6%) | 3.5 (1) | NA | |
| **Pregnant or breastfeeding women** | | | |
| Eligible N=1219 (25.8%) | 5.7 (70) | 278 (128) | NA |
| Ineligible N=29 (0.6%) | 6.9 (2) | NA | |

Of note, although relapse was less common in this cohort than in the main cohort (6.7% vs. 18.4% of ineligible relapsed within 12 months) and statistical power was limited, the relative differences between eligible and ineligible were in line with the main analyses.

© 2022 Taipale H et al. *JAMA Psychiatry.*

## Online-only Figures

### eFigure 1. Proportion of specific antipsychotics used by individuals eligible vs ineligible for randomised controlled trials in the Finnish and Swedish cohorts

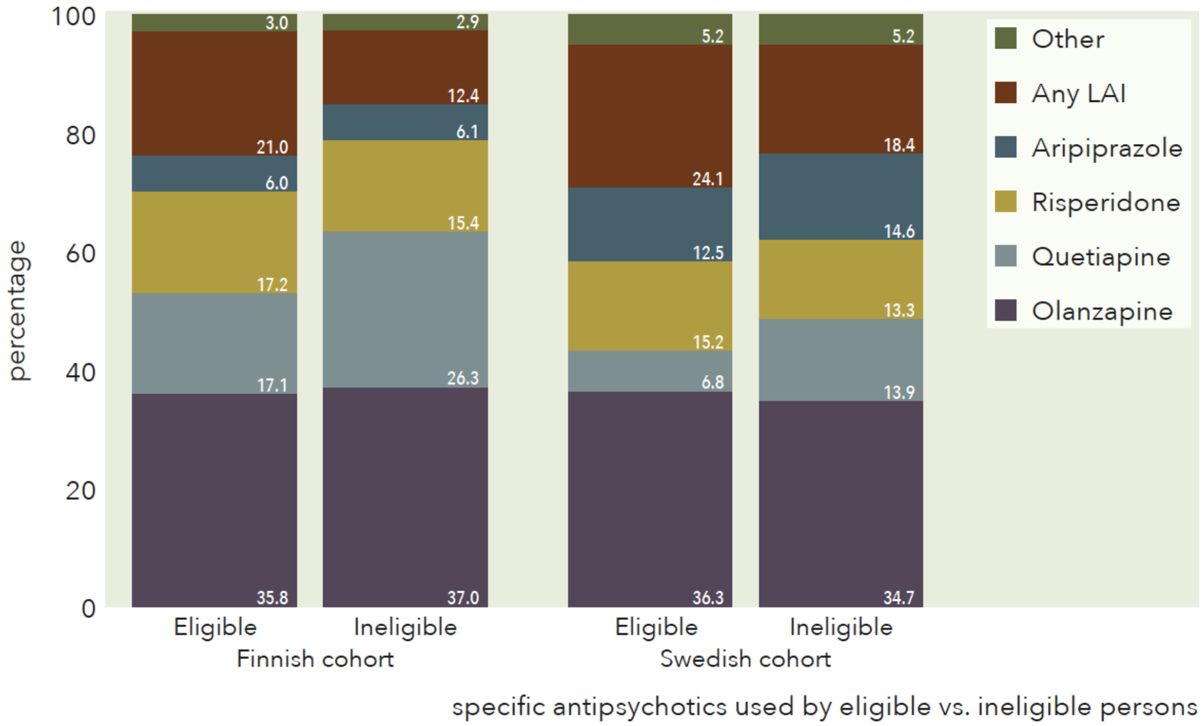


© 2022 Taipale H et al. *JAMA Psychiatry*.

**eFigure 2. Proportion of specific antipsychotics used within individuals ineligible due to specific reasons in the Finnish cohort.**

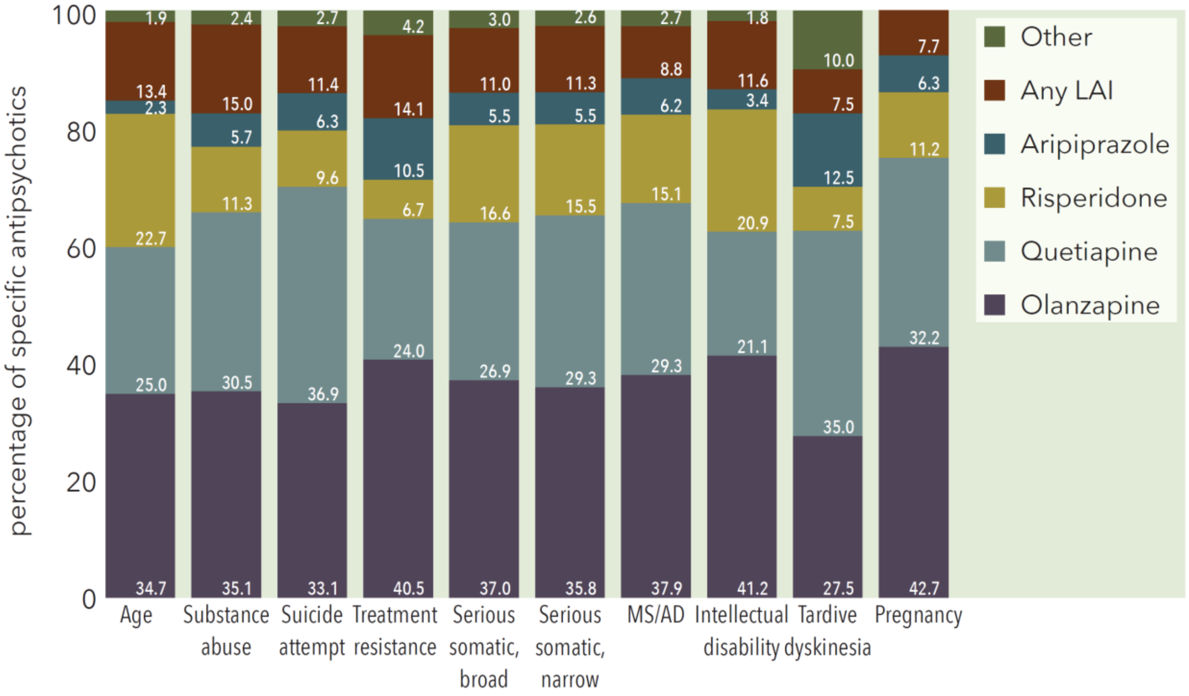


© 2022 Taipale H et al. *JAMA Psychiatry*.

**eFigure 3. Proportion of specific antipsychotics used within individuals ineligible due to specific reasons in the Swedish cohort**

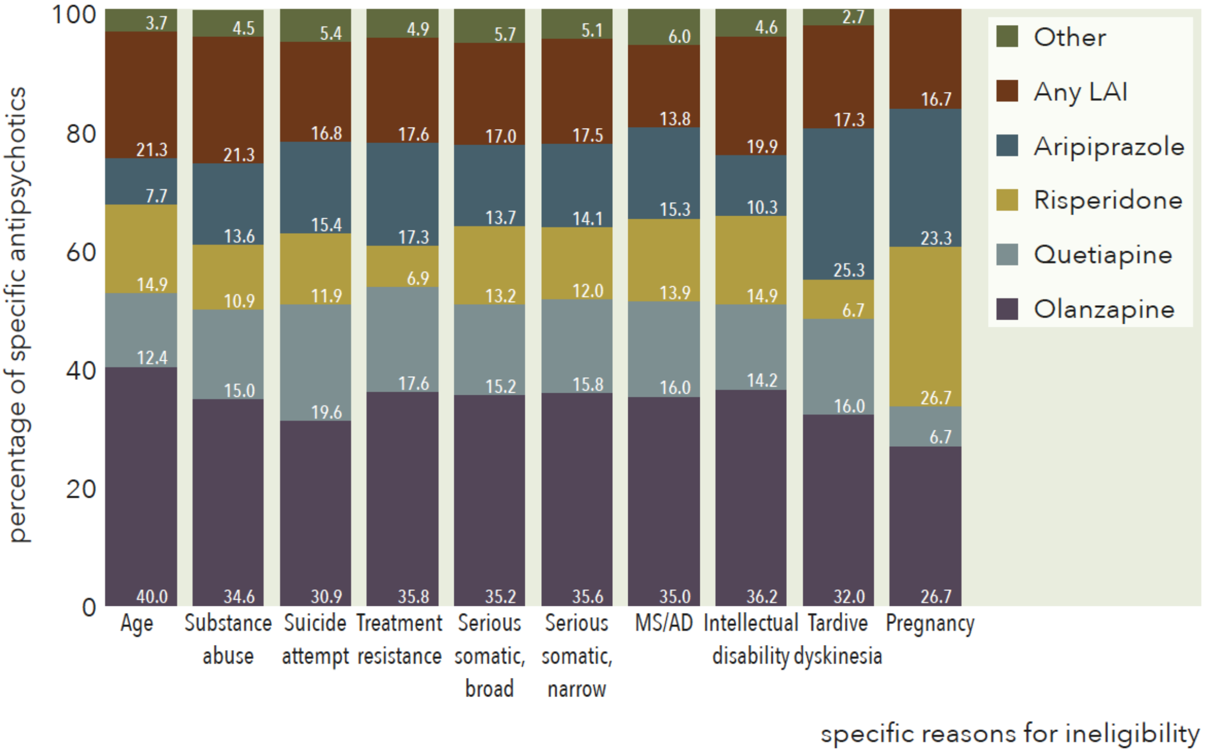


© 2022 Taipale H et al. *JAMA Psychiatry*.

**eAppendix 2. The RECORD statement – checklist of items, extended from the STROBE statement, that should be reported in observational studies using routinely collected health data.**

| | Item No. | STROBE items | Location in manuscript where items are reported | RECORD items | Location in manuscript where items are reported |
|---|---|---|---|---|---|
| **Title and abstract** | | | | | |
| | 1 | (a) Indicate the study's design with a commonly used term in the title or the abstract (b) Provide in the abstract an informative and balanced summary of what was done and what was found | Pages 1 and 3. | RECORD 1.1: The type of data used should be specified in the title or abstract. When possible, the name of the databases used should be included.<br><br>RECORD 1.2: If applicable, the geographic region and timeframe within which the study took place should be reported in the title or abstract.<br><br>RECORD 1.3: If linkage between databases was conducted for the study, this should be clearly stated in the title or abstract. | Page 3.<br><br>Page 3. |
| **Introduction** | | | | | |
| Background rationale | 2 | Explain the scientific background and rationale for the investigation being reported | Page 5. | | |
| Objectives | 3 | State specific objectives, including any prespecified hypotheses | Page 5., Abstract/Objectives, Key Points/Question | | |
| **Methods** | | | | | |
| Study Design | 4 | Present key elements of study design early in the paper | Page 6. | | |
| Setting | 5 | Describe the setting, locations, and relevant dates, including periods of recruitment, exposure, follow-up, and data collection | Page 6. | | |
| Participants | 6 | *(a) Cohort study* - Give the eligibility criteria, and the sources and methods of selection of | | RECORD 6.1: The methods of study population selection (such as codes or algorithms used to identify subjects) should be listed in detail. If this is not | Page 6; eMethods page 4-6. |

© 2022 Taipale H et al. *JAMA Psychiatry*.

| | | | | | |
|---|---|---|---|---|---|
| | | participants. Describe methods of follow-up<br>*Case-control study* - Give the eligibility criteria, and the sources and methods of case ascertainment and control selection. Give the rationale for the choice of cases and controls<br>*Cross-sectional study* - Give the eligibility criteria, and the sources and methods of selection of participants<br><br>*(b) Cohort study* - For matched studies, give matching criteria and number of exposed and unexposed<br>*Case-control study* - For matched studies, give matching criteria and the number of controls per case | | possible, an explanation should be provided.<br><br>RECORD 6.2: Any validation studies of the codes or algorithms used to select the population should be referenced. If validation was conducted for this study and not published elsewhere, detailed methods and results should be provided.<br><br>RECORD 6.3: If the study involved linkage of databases, consider use of a flow diagram or other graphical display to demonstrate the data linkage process, including the number of individuals with linked data at each stage. | |
| Variables | 7 | Clearly define all outcomes, exposures, predictors, potential confounders, and effect modifiers. Give diagnostic criteria, if applicable. | Page 7-9 | RECORD 7.1: A complete list of codes and algorithms used to classify exposures, outcomes, confounders, and effect modifiers should be provided. If these cannot be reported, an explanation should be provided. | |
| Data sources/ measurement | 8 | For each variable of interest, give sources of data and details of methods of assessment (measurement).<br>Describe comparability of assessment methods if there is more than one group | Pages 7 – 8 | | |
| Bias | 9 | Describe any efforts to address potential sources of bias | Pages 14-15 | | |
| Study size | 10 | Explain how the study size was arrived at | Page 10 | | |
| Quantitative variables | 11 | Explain how quantitative variables were handled in the analyses. If | Pages 7 – 9 ;<br>eMethods page 4. | | |

© 2022 Taipale H et al. *JAMA Psychiatry*.

| | | | | | |
|---|---|---|---|---|---|
| | | applicable, describe which groupings were chosen, and why | | | |
| Statistical methods | 12 | (a) Describe all statistical methods, including those used to control for confounding<br>(b) Describe any methods used to examine subgroups and interactions<br>(c) Explain how missing data were addressed<br>(d) *Cohort study* - If applicable, explain how loss to follow-up was addressed<br>*Case-control study* - If applicable, explain how matching of cases and controls was addressed<br>*Cross-sectional study* - If applicable, describe analytical methods taking account of sampling strategy<br>(e) Describe any sensitivity analyses | Pages 7 – 9; eMethods page 4 – 6. | | |
| Data access and cleaning methods | | .. | | RECORD 12.1: Authors should describe the extent to which the investigators had access to the database population used to create the study population.<br><br>RECORD 12.2: Authors should provide information on the data cleaning methods used in the study. | Pages 7 – 9; online-only eMethods page 4 – 6. |
| Linkage | | .. | | RECORD 12.3: State whether the study included person-level, institutional-level, or other data linkage across two or more databases. The methods of linkage and methods of linkage quality evaluation should be provided. | |
| **Results** | | | | | |
| Participants | 13 | (a) Report the numbers of individuals at each stage of the | Page 10. | RECORD 13.1: Describe in detail the selection of the persons included in the | Pages 10 – 12; online-only |

© 2022 Taipale H et al. *JAMA Psychiatry*.

| | | | | | |
|---|---|---|---|---|---|
| | | study (*e.g.*, numbers potentially eligible, examined for eligibility, confirmed eligible, included in the study, completing follow-up, and analysed)<br>(b) Give reasons for non-participation at each stage.<br>(c) Consider use of a flow diagram | | study (*i.e.,* study population selection) including filtering based on data quality, data availability and linkage. The selection of included persons can be described in the text and/or by means of the study flow diagram. | eMethods page 4-6. |
| Descriptive data | 14 | (a) Give characteristics of study participants (*e.g.*, demographic, clinical, social) and information on exposures and potential confounders<br>(b) Indicate the number of participants with missing data for each variable of interest<br>(c) *Cohort study* - summarise follow-up time (*e.g.*, average and total amount) | Pages 10 – 12, Table 1 | | |
| Outcome data | 15 | *Cohort study* - Report numbers of outcome events or summary measures over time<br>*Case-control study* - Report numbers in each exposure category, or summary measures of exposure<br>*Cross-sectional study* - Report numbers of outcome events or summary measures | Pages 11 – 12. | | |
| Main results | 16 | (a) Give unadjusted estimates and, if applicable, confounder-adjusted estimates and their precision (e.g., 95% confidence interval). Make clear which confounders were adjusted for and why they were included<br>(b) Report category boundaries when continuous variables were categorized | Pages 10 – 13; 25 – 27. | | |

© 2022 Taipale H et al. *JAMA Psychiatry*.

| | | | | | |
|---|---|---|---|---|---|
| | | (c) If relevant, consider translating estimates of relative risk into absolute risk for a meaningful time period | | | |
| Other analyses | 17 | Report other analyses done—e.g., analyses of subgroups and interactions, and sensitivity analyses | Page 12; online-only supplement page 5, 36-45 | | |
| **Discussion** | | | | | |
| Key results | 18 | Summarise key results with reference to study objectives | Page 14. | | |
| Limitations | 19 | Discuss limitations of the study, taking into account sources of potential bias or imprecision. Discuss both direction and magnitude of any potential bias | Page 15 – 16; | RECORD 19.1: Discuss the implications of using data that were not created or collected to answer the specific research question(s). Include discussion of misclassification bias, unmeasured confounding, missing data, and changing eligibility over time, as they pertain to the study being reported. | Page 14. |
| Interpretation | 20 | Give a cautious overall interpretation of results considering objectives, limitations, multiplicity of analyses, results from similar studies, and other relevant evidence | Pages 14 – 16. | | |
| Generalisability | 21 | Discuss the generalisability (external validity) of the study results | Page 15 | | |
| **Other Information** | | | | | |
| Funding | 22 | Give the source of funding and the role of the funders for the present study and, if applicable, for the original study on which the present article is based | Pages 18 – 19 | | |
| Accessibility of protocol, raw data, and programming code | | .. | | RECORD 22.1: Authors should provide information on how to access any supplemental information such as the | Page 7 |

© 2022 Taipale H et al. *JAMA Psychiatry*.

| | | | | | |
|---|---|---|---|---|---|
| | | | | study protocol, raw data, or programming code. | |

*Reference: Benchimol EI, Smeeth L, Guttmann A, Harron K, Moher D, Petersen I, Sørensen HT, von Elm E, Langan SM, the RECORD Working Committee. The REporting of studies Conducted using Observational Routinely-collected health Data (RECORD) Statement. *PLoS Medicine* 2015; in press. *Checklist is protected under Creative Commons Attribution (CC BY) license.

© 2022 Taipale H et al. *JAMA Psychiatry*.
